# Supplementary material for: Substantial but spatially heterogeneous progress in male circumcision for HIV prevention in South Africa
Source: Commun Med (Lond). 2024 Jan 3;4:1. doi: 10.1038/s43856-023-00405-7 (PMC10764768; doi:10.1038/s43856-023-00405-7)
Supplement: Supplementary file 2 — Supplementary Information [file 43856_2023_405_MOESM2_ESM.pdf]

Supplementary Information

Substantial but spatially heterogeneous progress in male circumcision for HIV prevention in South Africa

Matthew L. Thomas[1,2], Khangelani Zuma[3,4], Dayanund Loykissoonlal[5], Ziphozonke Bridget Dube[6], Peter Vranken[7], Sarah E. Porter[7], Katharine Kripke[8], Thapelo Seatlhodi[5,9], Gesine Meyer-Rath[10,11], Leigh F. Johnson[9], Jeffrey W. Imai-Eaton[2,12]

1 Department of Earth and Environmental Sciences, University of Manchester, Manchester, United Kingdom

2 MRC Centre for Global Infectious Disease Analysis, School of Public Health, Imperial College London, London, United Kingdom

3 Human and Social Capabilities Research Division, Human Sciences Research Council, Pretoria, South Africa

4 School of Public Health, University of the Witwatersrand, Johannesburg, South Africa

5 National Department of Health, Pretoria, South Africa

6 Genesis Analytics, Johannesburg, South Africa

7 Division of Global HIV and Tuberculosis, Centers for Disease Control and Prevention, Pretoria, South Africa

8 Avenir Health, Washington, United States of America

9 Centre for Infectious Disease Epidemiology and Research, University of Cape Town, Cape Town, South Africa

10 Health Economics and Epidemiology Research Office, Faculty of Health Sciences, University of Witwatersrand, Johannesburg, South Africa

11 Department of Global Health, Boston University School of Public Health, Boston, United States of America

12 Center for Communicable Disease Dynamics, Department of Epidemiology, Harvard T.H. Chan School of Public Health, Boston, United States of America

Supplementary Methods

Using a Bayesian hierarchical model with small area estimation methods, we modelled probabilities of circumcision stratified by region, age, and time for two types of circumcision: (1) circumcisions that occurred in traditional male initiation ceremonies or for other religious or cultural reasons (TMIC) and (2) circumcisions for non-traditional reasons and/or HIV prevention that take place in a clinical setting using medical methods (MMC-nT). Reflecting recent efforts to encourage adoption of medical methods in TMICs, TMICs were sub-categorised into: (1) traditional circumcisions conducted using non-medical methods (TMC) and (2) circumcisions conducted as part of TMIC but using medical methods (MMC-T), determined by a district- and year-specific probability that TMIC circumcisions were performed as MMC-Ts.

Probabilities of circumcision were estimated using a competing risk discrete time-to-event model [1], with estimates of circumcision coverage by type within each cohort calculated using the cumulative incidence. These were combined with data on male population size to calculate the predicted number of circumcisions conducted per year. Likelihood functions were specified for the two data sources to inform parameter calibration: (1) the probability of individual-level observations of circumcision age, type, and year reported by men in national household surveys in a time-to-event framework, and (2) the reported number of medical male circumcisions conducted in each district for HIV prevention among males 10 years and older using a Poisson count model.

Throughout the methods and applications sections, we refer to the following types of circumcision (Supplementary Figure 1):

- **MMC-nT:** Medical male circumcisions conducted outside of traditional male initiation ceremonies, representing the large majority of MMC conducted.
- **TMC:** Traditional male circumcisions, assumed to be conducted outside a medical setting for traditional male initiation purposes.
- **MMC-T:** Medical male circumcisions conducted as part of traditional male initiation ceremonies, typically in place of circumcisions that previously would have been conducted as TMC.

Useful aggregates of these circumcision types are referred to as:

- **MMC:** All medical male circumcisions (MMC-nT + MMC-T), assumed to be consistent with circumcisions reported through VMMC programme data reporting.
- **TMIC:** All male circumcisions conducted as part of traditional male initiation practices (TMC + MMC-T).

- **MC:** All male circumcisions of any type (MMC-nT + TMC + MMC-T).

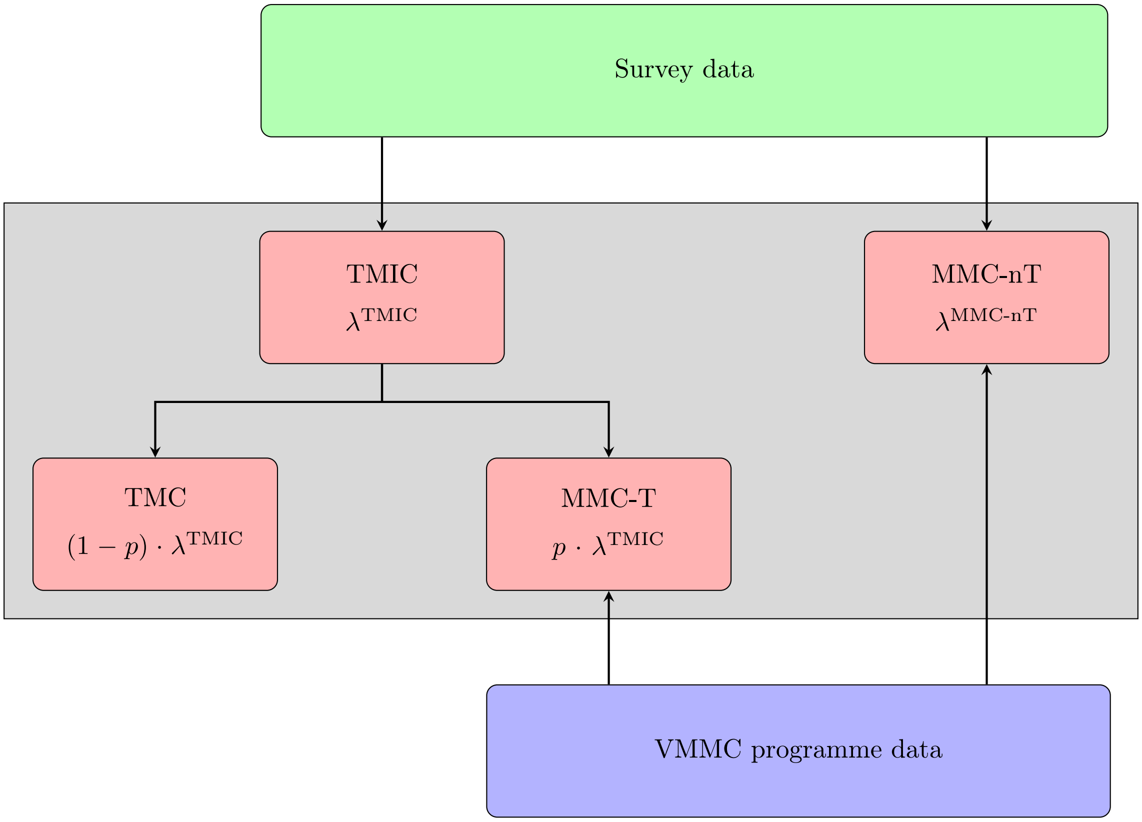

Supplementary Figure 1: Schematic representation of types of circumcision included in the model and data sources informing each. TMIC = circumcisions conducted as part of traditional male initiation ceremonies; TMC = traditional male circumcisions (conducted by a non-medical practitioner); MMC-T = medical male circumcision conducted as part of traditional male initiation practices; MMC-nT = medical male circumcision conducted outside the context of traditional male initiation (the majority of VMMC for HIV prevention and other routine medical male circumcision). The parameters $\lambda^{\text{MMC-nT}}$ and λ^{TMIC} represent the probabilities of having an MMC-nT and TMC respectively and p represent the proportion of TMICs conducted as medical methods, as described in Section .

Supplementary Table 1: Summary of model inputs and outputs.

<table>
<tr>
<td>

Model inputs

Area hierarchy. List of administrative areas used for health planning ("districts"), geographic boundaries, and nesting in higher level administrative areas.

Population. Population estimate stratified by district, sex, and single-year age group (0,1, $\cdots$, 59) covering the period of interest.

Household surveys. Data related to age, residence, (self-reported) circumcision status, age at circumcision and type of circumcision (medical/traditional) from recent HIV household surveys.

VMMC programme data. Data on number of VMMCs performed for HIV prevention by district and year.

</td>
<td>

Model outputs

Indicators. The model produces outputs for the following indicators:

- *Probabilities of circumcision*
- *Circumcision coverage (cumulative incidence)*
- *Male population*
- *Number circumcised*
- *Number uncircumcised*

Stratifications. All indicators are stratified according to:

- *Geographic*:
  - All levels of the area hierarchy (e.g. national, province, district)
- *Age groups*:
  - Single-year age group (0, 1, $\cdots$, 59)
  - Five-year age groups (0-4, 5-9, $\cdots$, 55-59)
  - Others (0+, 10+, 15+, 15-24, 10-24, 15-29, 10-29, 15-39, 10-39, 15-49, 10-49 and 25-49)
- *Type*:
  - MMC-nT
  - MMC-T
  - TMC
  - MMC (MMC-nT + MMC-T)
  - TMIC (TMC + MMC-T)
  - Total (MMC + MMC-T + TMC)
- *Time*:
  - Annual, 2008-2019

Statistics. Posterior summary statistics are computed for:

- Point estimates:
  - Mean
  - Median
- Uncertainty:
  - Standard deviation
  - 95% credible interval (quantile-based)

</td>
</tr>
</table>

Process model

Probabilities of circumcision per time step

Utilising data from household surveys, we modelled the probabilities of becoming circumcised per time step through either TMIC or MMC-nT for individuals residing in region $i \in I = \{1, 2, \dots, N_I\}$ having an MMC, MMC-nT, MMC-T, TMC, TMIC or MMC at age $a \in A = \{0, 1, 2, \dots, N_A\}$ and time $t \in T = \{1, 2, \dots, N_T\}$.

We defined $\lambda_{iat}^{\text{TMIC}}$ as the probability that an individual in region i received TMIC at age a and time step t, given they were uncircumcised by age $a-1$ and time $t-1$,

$$\lambda_{iat}^{\text{TMIC}} = \mathbb{P}(\text{TMIC in } (i, a, t) \mid \text{Uncircumcised in } (i, a-1, t-1)). \quad (1)$$

This was modelled using piece-wise logit-linear function:

$$\text{logit}(\lambda_{iat}^{\text{TMIC}}) = \hat{\alpha} + \hat{\psi}_i + \hat{\phi}_a + \hat{\gamma}_{ia}$$

where $\hat{\alpha}$ is the intercept, $\hat{\psi}_i$ is a regional random effect, $\hat{\phi}_a$ is an age random effect, and $\hat{\gamma}_{ia}$ is an age-space interaction term to allow different age patterns of TMIC across regions. We assumed that the probability of TMIC was constant over time (i.e. $\lambda_{iat}^{\text{TMIC}} \equiv \lambda_{ia}^{\text{TMIC}}$) as TMIC practices have been relatively stable over time.

Similarly, we defined $\lambda_{iat}^{\text{MMC-nT}}$ as the probability an individual in region i received MMC-nT at age a and time t, $\lambda_{iat}^{\text{MMC-T}}$, given they were uncircumcised by age $a-1$ and time $t-1$,

$$\lambda_{iat}^{\text{MMC-nT}} = \mathbb{P}(\text{MMC-nT in } (i, a, t) \mid \text{Uncircumcised in } (i, a-1, t-1)), \quad (2)$$

To ensure the overall probability of circumcision $\lambda_{iat}^{\text{TMIC}} + \lambda_{iat}^{\text{MMC-nT}} \leq 1$ for all i, a and t in the discrete-time framework, we applied the probability of TMIC before MMC-nT in each time step, and therefore initially model the probability an individual in region i received MMC-nT at age a and time t, $\lambda_{iat}^{\text{MMC-T}}$, given they were uncircumcised by age $a-1$ and time $t-1$ and did not recieve TMIC at age a and time t

$$\tilde{\lambda}_{iat}^{\text{MMC-nT}} = \mathbb{P}(\text{MMC-nT in } (i, a, t) \mid \text{Uncircumcised in } (i, a-1, t-1) \text{ and no TMIC in } (i, a, t))$$

such that

$$\lambda_{iat}^{\text{MMC-nT}} = \tilde{\lambda}_{iat}^{\text{MMC-nT}} \cdot (1 - \lambda_{iat}^{\text{TMIC}}).$$

The probability $\tilde{\lambda}_{iat}^{\text{MMC-nT}}$ was separated into two processes: (i) paediatric circumcision (for those aged 0–9) and (ii) adolescent and adult circumcision (for those aged 10 and over). VMMC programmes only provide MMCs for HIV prevention to those aged 10 and over, while infant and paediatric medical circumcision tends to occur through cultural or religious practices unrelated to scale-up of VMMC for HIV prevention. Therefore, we modelled $\tilde{\lambda}_{iat}^{\text{MMC-nT}}$ using a piece-wise logit-linear function:

$$\text{logit}(\tilde{\lambda}_{iat}^{\text{MMC-nT}}) = \begin{cases} \bar{\alpha} + \bar{\psi}_i + \bar{\phi}_a + \bar{\gamma}_{ia} & \text{for } 0 \leq a \leq 9 \\ \alpha + \psi_i + \phi_a + \theta_t + \gamma_{ia} + \delta_{at} + \zeta_{it} & \text{for } a \geq 10 \end{cases}$$

where $\bar{\alpha}$ and α are intercepts, $\bar{\psi}_i$ and ψ_i are regional random effects, θ_i are temporal random effects, $\bar{\phi}_a$ and ϕ_a are age random effects, $\bar{\gamma}_{ia}$ and γ_{ia} are age-space interaction terms to allow for different age patterns of MMC-nTs between regions, for paediatric circumcision and adolescent and adult circumcision respectively. The δ_{at} is an age-time interaction term to allow for different age patterns of MMC-nTs over time and ζ_{it} is an space-time interaction term to allow for different uptake of MMC-nTs over time across regions, in adolescent and adult men. Similarly, we assumed that the probabilities of MMC-nT among those aged 0–9 were constant over time, (i.e. $\lambda_{iat}^{\text{MMC-nT}} \equiv \lambda_{ia}^{\text{MMC-nT}}$ when $0 \leq a \leq 9$), as paediatric medical circumcision practices are unrelated to VMMC for HIV prevention and are relatively stable.

For individuals circumcised in TMIC, we defined a probability, p_{iat} that circumcision was conducted as a MMC-T versus TMC, specified for region i, age a, and time t. The probability an individual in region i received MMC-T at age a and time t, $\lambda_{iat}^{\text{MMC-T}}$ was thus

$$\begin{aligned} \lambda_{iat}^{\text{MMC-T}} &= \mathbb{P}(\text{MMC-T in } (i, a, t) \mid \text{Uncircumcised in } (i, a-1, t-1)) \\ &= p_{iat} \cdot \lambda_{iat}^{\text{TMIC}} \end{aligned} \tag{3}$$

and the probability an individual in region i received TMIC was

$$\begin{aligned} \lambda_{iat}^{\text{TMC}} &= \mathbb{P}(\text{TMC in } (i, a, t) \mid \text{Uncircumcised in } (i, a-1, t-1)) \\ &= (1 - p_{iat}) \cdot \lambda_{iat}^{\text{TMIC}} \end{aligned} \tag{4}$$

Prior to VMMC programmes, all circumcisions conducted in TMIC were TMC, in which case $p_{iat} = 0$. The proportion of TMICs conducted as MMC-Ts may not be identifiable from the survey data, particularly for years since the most recent survey. Thus $p_{iat} > 0$ should only be introduced in regions where there is knowledge that MMC-Ts were implemented in TMIC settings.

Taken together, the probability of circumcision of any type, at age a and time t given they were

uncircumcised by age $a-1$ and time $t-1$ was

$$\begin{aligned}\lambda_{iat}^{\text{MC}} &= \mathbb{P}(\text{MC in } (i,a,t) \mid \text{Uncircumcised in } (i,a-1,t-1)) \\ &= \lambda_{iat}^{\text{TMC}} + \lambda_{iat}^{\text{MMC-T}} + \lambda_{iat}^{\text{MMC-nT}} \\ &= \lambda_{ia}^{\text{TMIC}} + \lambda_{iat}^{\text{MMC-nT}}.\end{aligned} \tag{5}$$

The probability an individual in region i remained uncircumcised at age a and time t, given they were uncircumcised by age $a-1$ and time $t-1$, was

$$\begin{aligned}\lambda_{iat}^{\text{UC}} &= \mathbb{P}(\text{Uncircumcised in } (i,a,t) \mid \text{Uncircumcised in } (i,a-1,t-1)), \\ &= 1 - \lambda_{iat}^{\text{TMIC}} - \lambda_{iat}^{\text{MMC-nT}} \\ &= (1 - \lambda_{iat}^{\text{TMIC}})(1 - \tilde{\lambda}_{iat}^{\text{MMC-nT}})\end{aligned} \tag{6}$$

Cumulative incidence of circumcision

The probability S_{iat} of remaining uncircumcised up to age a and time t, typically referred to as the survivor function, was expressed as

$$\begin{aligned}S_{iat} &= \mathbb{P}(\text{Uncircumcised in } (i,a-1,t-1)) \\ &= \prod_{(0,(t-a))}^{(a-1,t-1)} \lambda_{iat}^{\text{UC}} \\ &= \lambda_{i,0,t-a}^{\text{UC}} \cdot \lambda_{i,1,t-a+1}^{\text{UC}} \cdot \ldots \cdot \lambda_{i,a-1,t-1}^{\text{UC}}\end{aligned} \tag{7}$$

The cumulative incidence function (CIF) defines the marginal probability (or proportion/coverage of) individuals who were circumcised by type $k \in K = \{$ MMC, MMC-nT, MMC-T, TMC, TMIC or MMC$\}$ by age a and time t, accounting for the competing risk of other circumcision type. This was calculated as the sum of incidence of circumcision by type k at each age up to a:

$$\begin{aligned}\text{CIF}_{iat}^{k} &= \mathbb{P}(k \text{ by } (i,a,t)) \\ &= \sum_{(0,(t-a))}^{(a,t)} I_{iat}^{k}\end{aligned} \tag{8}$$

where I_{iat}^{k} is the incidence of circumcision type k in region i, age a and time t. For types $k \in \{\text{TMIC}, \text{TMC}, \text{MMC-T}\}$, this was defined by the probability of remaining uncircumcised by any type

up to age a at time t times the probability of circumcision by type k in year t at age a

$$\begin{aligned} I^k_{iat} &= \mathbb{P}(k \text{ in } (i,a,t)) \\ &= \mathbb{P}(k \text{ in } (i,a,t) | \text{ Uncircumcised in } (i,a-1,t-1)) \times \\ &\qquad \mathbb{P}(\text{Uncircumcised in } (i,a-1,t-1)) \\ &= \lambda^k_{iat} \cdot S_{iat} \end{aligned} \tag{9}$$

The overall incidence and CIF by any circumcision type can be obtained by summing across all circumcision types. The CIF is also often referred to as the 'sub-distribution function', due to the fact that the cumulative probability of a particular event k by time t will remain below one [1].

Programme data model

Utilising data on the number of VMMCs reported, we further informed the probabilities of becoming circumcised by region, age and time step. VMMCs conducted by public health programmes consisted of the total number of MMCs (both MMC-nT and MMC-T) conducted in region i at time t and age group $G = [a_1, a_2]$, Y_{iGt}. We modelled number of MMCs, $Y^{\text{MMC-nT}}_{iat}$, and MMC-Ts, $Y^{\text{MMC-T}}_{iat}$ in region i, at age a and time t using a Poisson likelihood with means μ^{MMC}_{iat} and $\mu^{\text{MMC-T}}_{iat}$ respectively,

$$Y^k_{iat} \mid \mu^k_{iat} \sim \text{Poisson}(\mu^k_{iat})$$

where k is MMC-T or MMC-nT. The mean μ^k_{iat} is determined by multiplying the male population size in region i, at age a and time t, P_{iat}, the probability of remaining uncircumcised in region i, up to age a and time t, S_{iat}, and the probability of being having a MMC-T or MMC-nT at age a and time t given they were uncircumcised by age $a-1$ and time $t-1$, $\lambda^{\text{MMC-T}}_{iat}$ or $\lambda^{\text{MMC-T}}_{iat}$

$$\mu^k_{iat} = P_{iat} \cdot S_{iat} \cdot \lambda^k_{iat}.$$

where k is MMC-T or MMC-nT. It then follows that the total number of MMCs conducted in region i at time t and age group $G = [a_1, a_2]$, Y_{iGt} are modelled using

$$\begin{aligned} Y_{iGt} \mid \mu_{iat} &\sim \text{Poisson}(\mu_{iGt}) \\ \mu_{iGt} &= \sum_{a=a_1}^{a_2} \mu^{\text{MMC-nT}}_{iat} + \mu^{\text{MMC-T}}_{iat}. \end{aligned}$$

Priors

In space, we assigned the random effects $\hat{\psi}_i$, $\bar{\psi}_i$, ψ_i, $\hat{\gamma}_{ia}$, γ_{ia} and ζ_{it}, intrinsic conditional autoregressive (ICAR) priors [2]. An ICAR model encodes spatial dependence between neighbours, defined as areas that share a common boundary, and allows information to be borrowed across regions, which may be useful in areas where data is sparse or non-existent. For a generic parameter β_i, an ICAR model assumes that the expected value of β_i is a weighted average its neighbours,

$$\beta_i \mid \beta_j, j \sim i, \tau_\beta \sim \mathrm{N}\left(\frac{1}{n_i}\sum_{j\sim i}\beta_j, \frac{1}{n_i\tau_\beta}\right) \quad i = 1, 2, 3, \ldots, N_I$$

where $j \sim i$ refers to the neighbours of region i, n_i is the number of neighbours and τ_β is the marginal precision. The joint distribution may be equivalently expressed as $\boldsymbol{\beta} \sim N(\mathbf{0}, \tau_\beta^{-1}Q_I^{-1})$ where Q_I is the precision matrix encoding the adjacency structure of the neighbourhoods. The precision matrix Q_I is rank deficient and consequently the prior is improper [3].

In time, we assigned the random effects θ_t, δ_{at} and ζ_{it} an autoregressive process of order 1 (AR1) priors,

$$\begin{aligned}\beta_t \mid \beta_{t-1}, \rho_\beta, \tau_\beta &\sim N(\rho_\beta \cdot \beta_{t-1},\ \ \tau_\beta^{-1}) \quad t = 2, 3, \ldots, N_T \\ \beta_1 \mid \rho_\beta, \tau_\beta &\sim N(0, \tau_\beta^{-1}) \quad t = 1\end{aligned}$$

where $|\rho_\beta| < 1$ is an autocorrelation parameters controlling the correlation of the effect of the current age on the previous age and τ_β is the precision. The joint distribution may be equivalently expressed as $\boldsymbol{\beta} \mid \rho_\beta, \tau_\beta \sim N(\mathbf{0}, \tau_\beta^{-1}Q_T^{-1}(\rho_\beta))$ where Q_T is the precision matrix and encodes the first order dependency in time controlled by the autocorrelation parameter ρ_β.

In age, we modelled the random effects $\hat{\phi}_a$, $\bar{\phi}_a$, ϕ_a, $\hat{\gamma}_{ia}$, $\bar{\gamma}_{ia}$, γ_{ia} and δ_{at} using penalised B-spline functions. For a generic parameter β_a, penalised B-splines assumes that β_a is a sum of basis functions,

$$\beta_a = \sum_{j=1}^{N_J} w_j b_{aj}$$

where b_{aj} are B-spline basis functions with knots placed every five years (at 0, 5, 10 etc.), N_J is the number of splines used and w_j are the spline weight parameters to be estimated. The weights w_j are

penalised using an AR1 process

$$
\begin{aligned}
w_j \mid w_{j-1}, \rho_w, \tau_w &\sim N(\rho_w \cdot w_{j-1},\ \ \tau_w^{-1}) \quad j = 2, 3, \ldots, N_J \\
w_1 \mid \rho_w, \tau_w &\sim N(0, \tau_w^{-1})
\end{aligned}
$$

where $|\rho_w| < 1$ is an autocorrelation parameters controlling the correlation of the effect of the current age on the previous age and τ_w is the precision. The joint distribution is given by $\boldsymbol{w} \mid \rho_w, \tau_w \sim N(\mathbf{0}, \tau_w^{-1} Q_A^{-1}(\rho_w))$ where Q_A is the precision matrix as defined by the smoothed function. Then defining $\boldsymbol{\beta} = W_\beta \cdot \boldsymbol{w}$ where W_β is a design matrix evaluating the basis functions f_{aj} at each age of interest, the joint distribution of $\boldsymbol{\beta} \mid \boldsymbol{w},\ W_\beta,\ \rho_w, \tau_w$ is given by, $\boldsymbol{\beta} \mid \boldsymbol{\omega},\ W_\beta,\ \rho_w, \tau_w \sim N(\mathbf{0}, \tau_w^{-1}(W_\beta \cdot Q_A^{-1}(\rho_w) \cdot W_\beta^T))$.

Moreover, the age-space, age-time, and space-time interaction terms ($\hat{\gamma}_{ia},\ \bar{\gamma}_{ia},\ \gamma_{ia},\ \delta_{at}$ and ζ_{it}) are modelled as Type IV interactions as defined by Knorr-Held and Leonard [4],

$$
\begin{aligned}
\hat{\boldsymbol{\gamma}} \mid \rho_{\hat{\gamma}}, \tau_{\hat{\gamma}} &\sim N(\mathbf{0},\ \tau_{\hat{\gamma}}^{-1} Q_{\hat{\gamma}}^{-1}) \\
\bar{\boldsymbol{\gamma}} \mid \rho_{\bar{\gamma}}, \tau_{\bar{\gamma}} &\sim N(\mathbf{0},\ \tau_{\bar{\gamma}}^{-1} Q_{\bar{\gamma}}^{-1}) \\
\boldsymbol{\gamma} \mid \rho_{\gamma}, \tau_{\gamma} &\sim N(\mathbf{0},\ \tau_{\gamma}^{-1} Q_{\gamma}^{-1}) \\
\boldsymbol{\delta} \mid \rho_{1\delta}, \rho_{2\delta}, \tau_{\delta} &\sim N(\mathbf{0},\ \tau_{\delta}^{-1} Q_{\delta}^{-1}) \\
\boldsymbol{\zeta} \mid \rho_{\zeta}, \tau_{\zeta} &\sim N(\mathbf{0},\ \tau_{\zeta}^{-1} Q_{\zeta}^{-1})
\end{aligned}
$$

where the precision matrices constructed using Kronecker products

$$
\begin{aligned}
Q_{\hat{\gamma}} &= [W_{\hat{\gamma}} \cdot Q_A(\rho_{\hat{\gamma}}) \cdot W_{\hat{\gamma}}^T] \otimes Q_I \\
Q_{\bar{\gamma}} &= [W_{\bar{\gamma}} \cdot Q_A(\rho_{\bar{\gamma}}) \cdot W_{\bar{\gamma}}^T] \otimes Q_I \\
Q_{\gamma} &= [W_{\gamma} \cdot Q_A(\rho_{\gamma}) \cdot W_{\gamma}^T] \otimes Q_I \\
Q_{\delta} &= [W_{\delta} \cdot Q_A(\rho_{1\delta}) \cdot W_{\delta}^T] \otimes Q_T(\rho_{2\delta}) \\
Q_{\zeta} &= Q_I \otimes Q_T(\rho_{\zeta})
\end{aligned}
$$

Here, Q_I, Q_A and Q_T are precision matrices discussed above.

Gaussian priors were assigned to each of the intercepts, $\alpha,\ \hat{\alpha},\ \bar{\alpha} \sim N(0, 5^2)$. Exponential priors were placed on standard deviations, $\sigma_{(\cdot)} = \sqrt{\tau_{(\cdot)}^{-1}} \sim \text{Exp}(1)$. Gaussian priors were set for all correlation

parameters on the logit scale, such that

$$\hat{\rho}_{(\cdot)} = \frac{2}{1+\exp(-\rho_{(\cdot)})} - 1 \sim N(3, 1^2)$$

corresponding to 95% CI prior weight that the autocorrelation parameters are between 0.48 and 0.99 on the real scale. The proportion of TMICs performed as MMC-Ts, p_{iat}, were assigned independent Gaussian distributions on the logit scale,

$$\text{logit}(p_{iat}) \sim N(\mu_{iat}, \sigma_{iat}^2)$$

if there is evidence or knowledge of the VMMC programmes to suggest MMC-Ts are taking place with some mean, μ_{iat}, and standard deviation, σ_{iat}. Otherwise, the proportions of TMICs performed as MMC-Ts are fixed at zero (p_{iat}=0).

Accounting for survey weights

The likelihood function consists of the product of the likelihoods for two independent data sources: (1) household survey data on individual male's age at circumcision and circumcision type, and (2) VMMC programme data about the number of circumcisions conducted for HIV prevention in each region.

For the former of these, survey data consists of individual observations of self-reported age at circumcision, date of birth, and circumcision type (medical or traditional) for male survey respondents. Circumcision status observations were right censored if the respondent reported not being circumcised at the time of the survey or left censored if the individual reported being circumcised at the time of survey but did not report their age at circumcision.

Each survey $s \in S = \{1, 2, \ldots, N_S\}$ consists of sampled individuals $j \in J_s = \{0, 1, 2, \ldots, N_{J_s}\}$ residing in region $i \in I = \{1, 2, \ldots, N_I\}$. Surveyed individuals were followed from their year of birth until their year of circumcision or their year of censoring. For circumcised individuals, the year of circumcision was calculated as the year of birth plus the age at circumcision. It was assumed that no circumcisions occurred after age 59, so for uncircumcised individuals, censoring occurred either in the survey year or when they were 59. Individuals who self-reported they were circumcised but have a missing age at circumcision were included in the analysis through left censoring.

The partial likelihood contribution for each individual is this defined by the following groups:

- **TMIC observed**: Individuals in region i reporting they were circumcised through TMI ceremonies aged a at time t. The likelihood of this outcome was the probability an individual in

region i reported a TMIC at age a and time t, $I_{iat}^{\text{TMIC}} = \lambda_{iat}^{\text{TMIC}} \cdot S_{iat}$.

- **MMC-nT observed**: Individuals in region i reporting they were medically circumcised aged a at time t. The likelihood of this outcome was the incidence of MMC-nT in region i, at age a and time t, $I_{iat}^{\text{MMC-nT}} = \lambda_{iat}^{\text{MMC-nT}} \cdot S_{iat}$.
- **Right censored**: Individuals in region i who were uncircumcised by age a at time t. The likelihood of this outcome was the probability of remaining uncircumcised in region i, at age a and time t,S_{iat}
- **Left censored**: Individuals in region i reporting a circumcision at an unknown age and time between birth at time $t-a$ and age a at time t. The likelihood of this outomce was the probability of being circumcised in region i before age a at time t, $(1 - S_{iat})$

Taken together, the partial likelihood for the survey data may be expressed as

$$\begin{aligned} L(\mathbf{\Theta}) &= \prod_{(i,a,t)} \prod_{s=1}^{N_s} \prod_{j=1}^{J_s} \underbrace{\left(\lambda_{iat}^{\text{TMIC}} \cdot S_{iat}\right)^{\mathbb{1}_{sjiat}^{\text{TMIC}}}}_{\text{TMIC observed}} \cdot \underbrace{\left(\lambda_{iat}^{\text{MMC-nT}} \cdot S_{iat}\right)^{\mathbb{1}_{sjiat}^{\text{MMC-nT}}}}_{\text{MMC-nT observed}} \cdot \underbrace{(S_{iat})^{\mathbb{1}_{sjiat}^{RC}}}_{\text{Right censored}} \cdot \underbrace{(1 - S_{iat})^{\mathbb{1}_{sjiat}^{LC}}}_{\text{Left censored}} \\ &= \prod_{(i,a,t)} \left(S_{iat}\lambda_{iat}^{\text{TMIC}}\right)^{\text{N}_{iat}^{\text{TMIC}}} \cdot \left(S_{iat}\lambda_{iat}^{\text{MMC-nT}}\right)^{\text{N}_{iat}^{\text{MMC-nT}}} \cdot (S_{iat})^{\text{N}_{iat}^{RC}} \cdot (1 - S_{iat})^{\text{N}_{iat}^{LC}} \end{aligned}$$

where $\mathbb{1}_{sjiat}^{l}$ is an indicator variable indicating whether individual j in survey s and region i reported outcome l at age a and time t. Here, N_{sjiat}^{l} denoting the total number of individuals reporting outcome l in region i at age a and time t

$$\text{N}_{iat}^{l} = \sum_{s=1}^{N_s} \sum_{j=1}^{J_s} \mathbb{1}_{sjiat}^{k}$$

where l is TMIC, MMC-nT, RC or LC.

Survey data are often collected through a complex two-stage cluster sampling design with unequal sampling probabilities. As a result, performing model inference using a standard likelihood may lead to biased estimates. To account for these survey designs, the probabilities of circumcision were estimated using a weighted pseudo-likelihood in which we replaced the observed counts N_{iat}^{l} with weighted counts $\tilde{\text{N}}_{iat}^{l}$, calculated using survey weights. Each individual j in survey s residing in region i had sampling weight ω_{sji} which were normalised using the Kish effective sample size,

$$\tilde{\omega}_{sji} = \frac{\omega_{sji}}{\bar{\omega}_{si}} \cdot \frac{M_s}{M_s^{\text{eff}}}$$

Here, $\bar{\omega}_{si}$ is the arithmetic mean of all survey weights from the individuals sampled in survey s and region i and M_s is the total sample size in survey s and M_s^{eff} is the Kish effective sample size which

accounts for heterogeneity in sampling weights and is calculated using

$$M_s^{\text{eff}} = \frac{(\sum_j \omega_{sji})^2}{\sum_j \omega_{sji}^2}.$$

Using the normalised sampling weights, $\tilde{\omega}_{sji}$, we calculate the weighted counts, $\tilde{\text{N}}_{iat}^{l}$, for each region, age and time stratum using

$$\tilde{\text{N}}_{iat}^{l} = \sum_{s=1}^{N_S} \sum_{j=1}^{N_{J_s}} \tilde{\omega}_{sji} \mathbb{1}_{sjiat}^{1}.$$

where l is either TMIC, MMC-nT, RC or LC.

Model checking

To assess the overall model fit, we performed posterior predictive checking for the full model (including both survey and VMMC programme data) and the model with only household survey data. We sampled 1000 values for medical or traditional circumcision status for each survey respondent based on predicted circumcision coverage prevalences by district-age-time strata. Both were aggregated to calculate predictive distributions by five year age groups (from 0-4 through 55-59).

We compared the mean predictions against the empirical survey estimates, using continuous ranked probability scores (CRPS), and error statistics such as the mean absolute error (ME) and root mean square error (RMSE). We also evaluated the coverage the posterior predictive distributions by calculating the proportion of empirical observations that fell within the 50%, 80%, and 95% quantiles of the posterior predictive distribution.

Inference

Models were implemented and fitted in R [5] using Template Model Builder (TMB) [6]. TMB is a software package that enables users to flexibly fit latent process/variable models to data. It uses automatic differentiation and Laplace approximations to estimate posterior distributions for model parameters. Models were optimised using the quasi-newton L-BFGS-B optimisation method [7].

The model estimates full posterior distributions of the annual probabilities of becoming circumcised and the corresponding circumcision coverage in South Africa between 2008 and 2019, by circumcision type, single-year age group, and district. Posterior predictive distributions for aggregates were obtained using Monte Carlo sampling. Samples were drawn from the joint posterior distributions, conditional on the optimised hyper-parameters [8], of the annual probabilities of becoming circumcised and the corresponding circumcision coverage in each region-age-time-type stratum. The joint samples were aggregated into any quantity of interest creating in a samples from the posterior distribution

of the quantity of interest. For each aggregate, the posterior mean, median, standard deviation, and quantile-based 95% credible intervals (CI) were computed from the corresponding posterior distribution. Samples were aggregated: (1) from district level to coarser administrative boundaries (province, national), (2) from single-year age groups to five-year age group (0–4, 5–9, etc.) and coarser priority age groups (15–49, 15–29, etc.) and (3) from individual types of circumcision (MMC-nT, MMC-T and TMC) to produce combinations including MMC, TMIC and MC. A full list of model outputs can be found in Supplementary Table 1.

Supplementary Notes 1

Survey data

Data from five nationally representative household surveys that asked men about their circumcision status conducted in South Africa between 2002 and 2017 were included in the analysis: South African National HIV Prevalence, HIV Incidence, Behaviour and Communication Survey (SABSSM) from 2002, 2008, 2012 and 2017 and the Demographic and Health Survey (DHS) from 2016. Information related to age, residence, self-reported circumcision status, age at circumcision, who performed the circumcision and where the circumcision took place were extracted for a total 51,261 male respondents across all surveys. Supplementary table 2 shows the availability of the questions related to circumcision.

Circumcisions were classed as either medical male circumcision for non-traditional reasons (MMC-nT) or circumcisions conducted in traditional male initiation ceremonies (TMIC) by using responses to both 'Who performed the circumcision?' and 'Where did the circumcision take place?' were classified as follows:

- Circumcisions that took place in a hospital or a clinic ***and*** were performed by a doctor, nurse or a healthcare worker were classified as MMC-nT.
- Circumcisions that took place at home, in a circumcision camp, in an initiation school, in the mountain or in the bush ***and*** were performed by spiritual leaders, religious leaders or a traditional circumciser were classified as TMIC.
- Circumcisions that took place at home, in a circumcision camp, in an initiation school, in the mountain or in the bush ***and*** were performed by doctors, nurses or a healthcare workers were classified as MMC-nT conducted as part of traditional male initiation (TMI) ceremonies.
- Circumcisions that took place in a hospital or a clinic ***and*** were performed by spiritual leaders, religious leaders or a traditional circumciser were classified as MMC-nT.

Individuals only with responses to ‘Who performed the circumcision?’ were classified as follows:

- Circumcisions performed by a doctor, nurse or a healthcare worker were classified as MMC-nT
- Circumcisions performed by spiritual leaders, religious leaders or a traditional circumciser were classified as TMIC

Individuals only with responses to ‘Where did the circumcision take place?’ were classified as follows:

- Circumcisions that took place in a hospital or a clinic were classified as MMC-nT
- Circumcisions that took place at home, in a circumcision camp, in an initiation school, in the mountain or in the bush were classified as TMIC

Individuals without responses to either question were excluded from the analysis. Supplementary Table 3 summarises the full criteria.

Supplementary Table 2: Availability of questions related to circumcision status extracted from the HSRC 2002, 2008, 2012 and 2017 surveys and the DHS 2016 survey.

	HSRC 2002	HSRC 2005	HSRC 2008	DHS 2016	HSRC 2017
Self-reported circumcision status	✓	✓	✓	✓	✓
Age at circumcision	✓	✓	✓	✓	✓
Who performed the circumcision?		✓	✓	✓	✓
Where did the circumcision take place?	✓	✓	✓		✓

Supplementary Table 3: Criteria used to classify circumcisions from the survey data as MMC-nT of TMIC.

		Who performed the circumcision?		
		Healthcare Professional	Spiritual/Religious Leaders	Missing†
Where performed?	Hospital or Clinic	MMC-nT	MMC-nT	MMC-nT
	Home, Camp, School or Bush	MMC-nT	TMIC	TMIC
	Missing††	MMC-nT	TMIC	Excluded

† Question unavailable in HSRC 2002 survey, †† Question unavailable in DHS 2016 survey

Programme data

The primary source of information on the reported number of VMMCs performed in South Africa between 2008-2019 was from the South Africa NDoH District Health Information System (DHIS) and supplemented with data from DMPPT2 and Datim. DHIS contains the number of VMMCs performed in each district in South Africa, reported on a monthly basis from March 2012 (for districts in KwaZulu-Natal), or April 2013 (for all other districts) through to October 2020. VMMCs were reported as ages 10+ until March 2017, and were reported for ages 10-14 and 15+ thereafter. DMPPT2 contains the number of VMMCs performed, reported annually (in South Africa financial years, April-March) between 2010 and 2017 in 5 year age groups (10-14, 15-19, 20-24, 25-29, 30-34, 35-39, 40-44, 45-49, 50-54 and 55-59). Datim contains the number of VMMCs performed by PEPFAR partners in 28 PEPFAR supported districts in South Africa, reported quarterly between April 2015 and October 2020. VMMCs were reported as ages 10-14, 15-19, 20-24, 25-29, 30-34, 35-39, 40-44, 45-49 and 50+, with ages 25-49, 30-49 and 40-49 used for some years. Supplementary Figures 2-4 show the number of circumcisions reported by DHIS, DMPPT2 and Datim respectively.

The number of VMMCs performed were aggregated to produce a number for men aged 10+ in each district for each financial year in South Africa, with data used from DMPPT2 for 2010-2012 and from DHIS for 2013-2017. For 2018 onwards, the number of VMMCs performed were estimated by combining data from DHIS and Datim. This is due large number of MMCs performed in a traditional setting in Xhosa men reported to PEPFAR in five Eastern Cape (EC) PEPFAR districts (Buffalo City, Amathole, Chris Hani, Alfred Nzo and Oliver Tambo) which were not reported to DHIS. An initial annual number of VMMCs performed for men aged 10+ for all districts, excluding the five EC PEPFAR districts, were extracted from DHIS.The number of VMMCs provided by PEPFAR in the five EC districts were then pooled and re-allocated to all districts in South Africa, according to the proportion of Xhosa men in each district. The total number of VMMCs performed in each district by data source can be seen in Supplementary Figure 5.

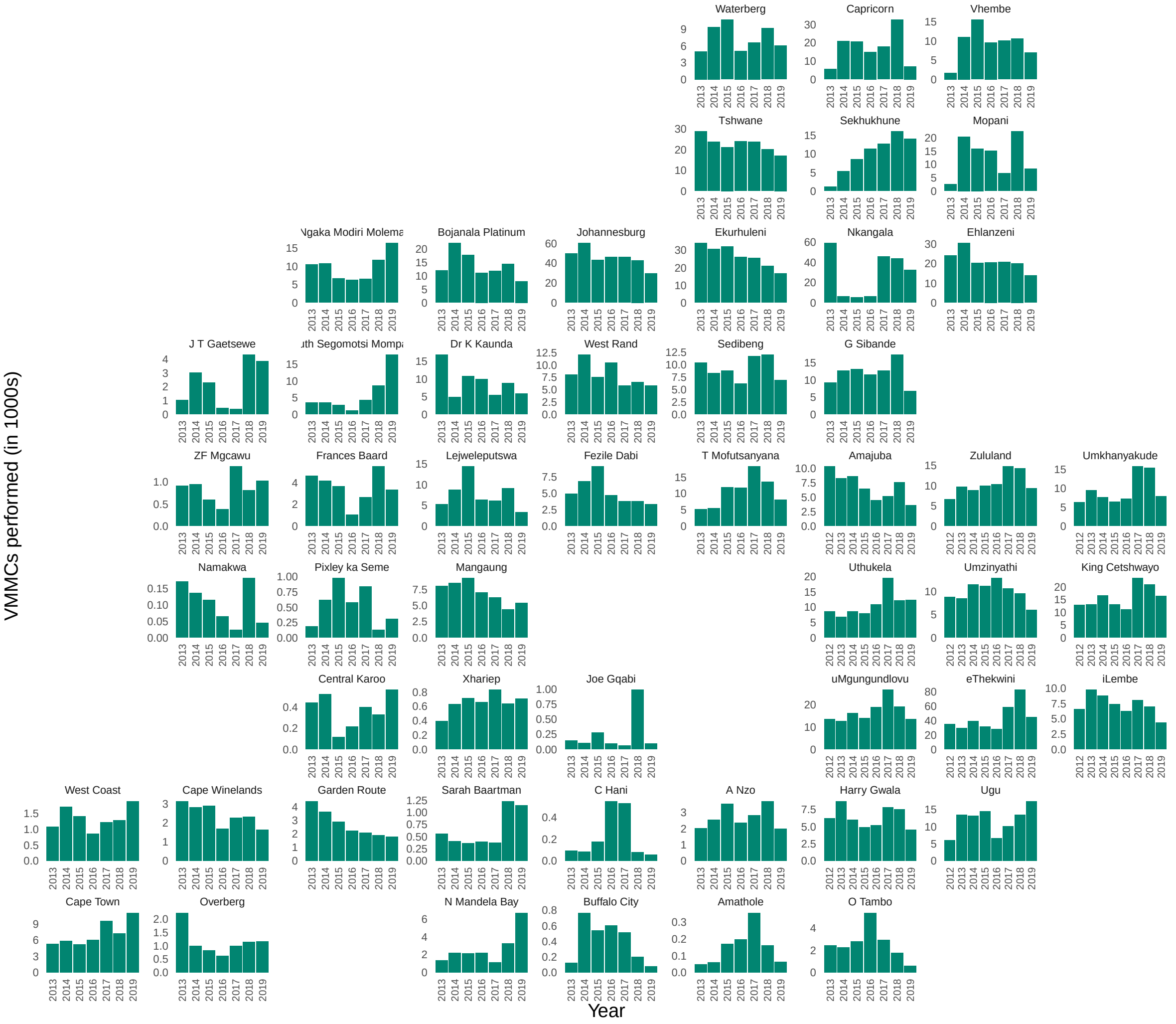

Supplementary Figure 2: Total number of VMMCs performed in each district between 2013 and 2019 reported to DHIS.

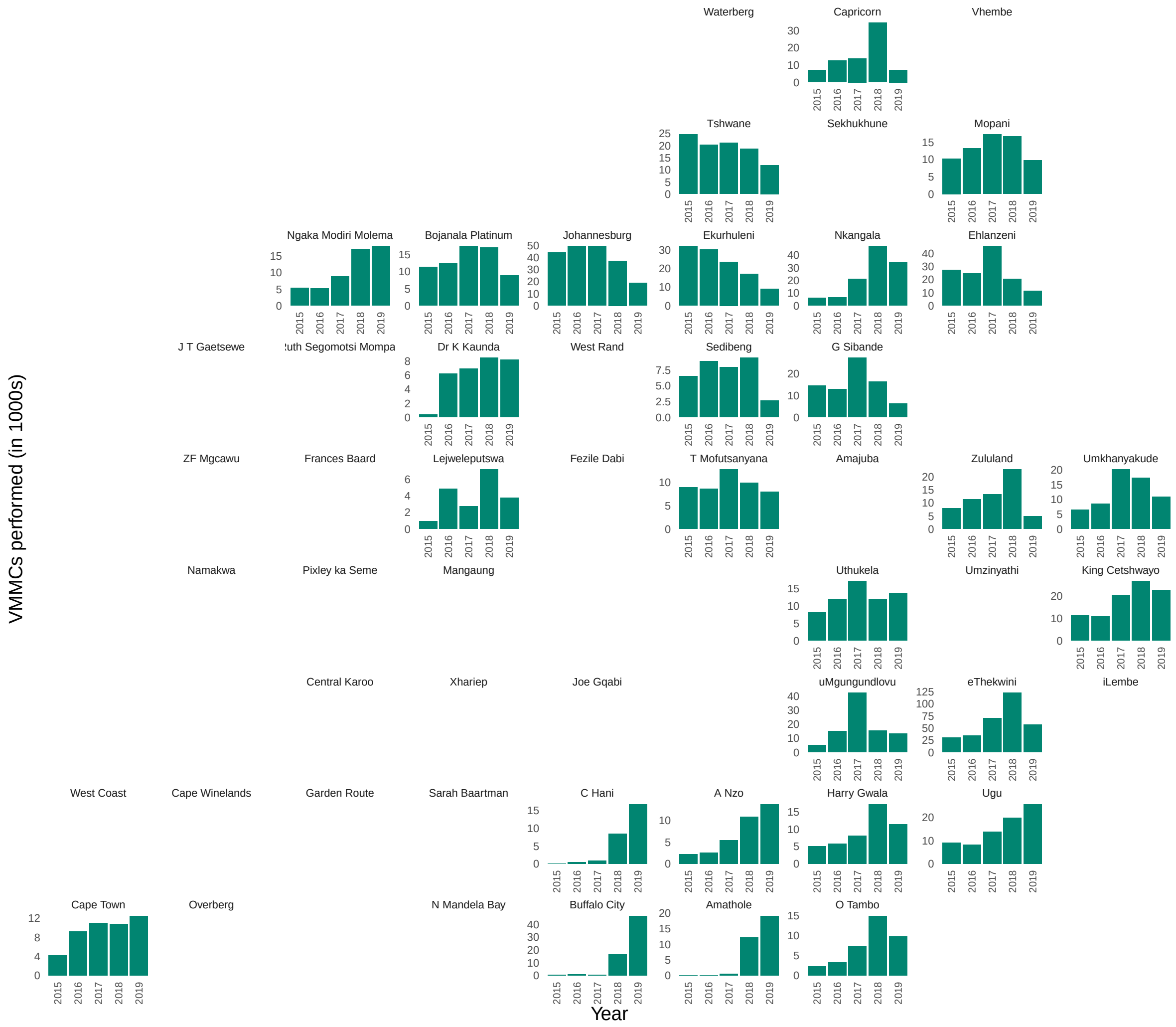

Supplementary Figure 3: Total number of VMMCs performed by PEPFAR between 2015 and 2019.

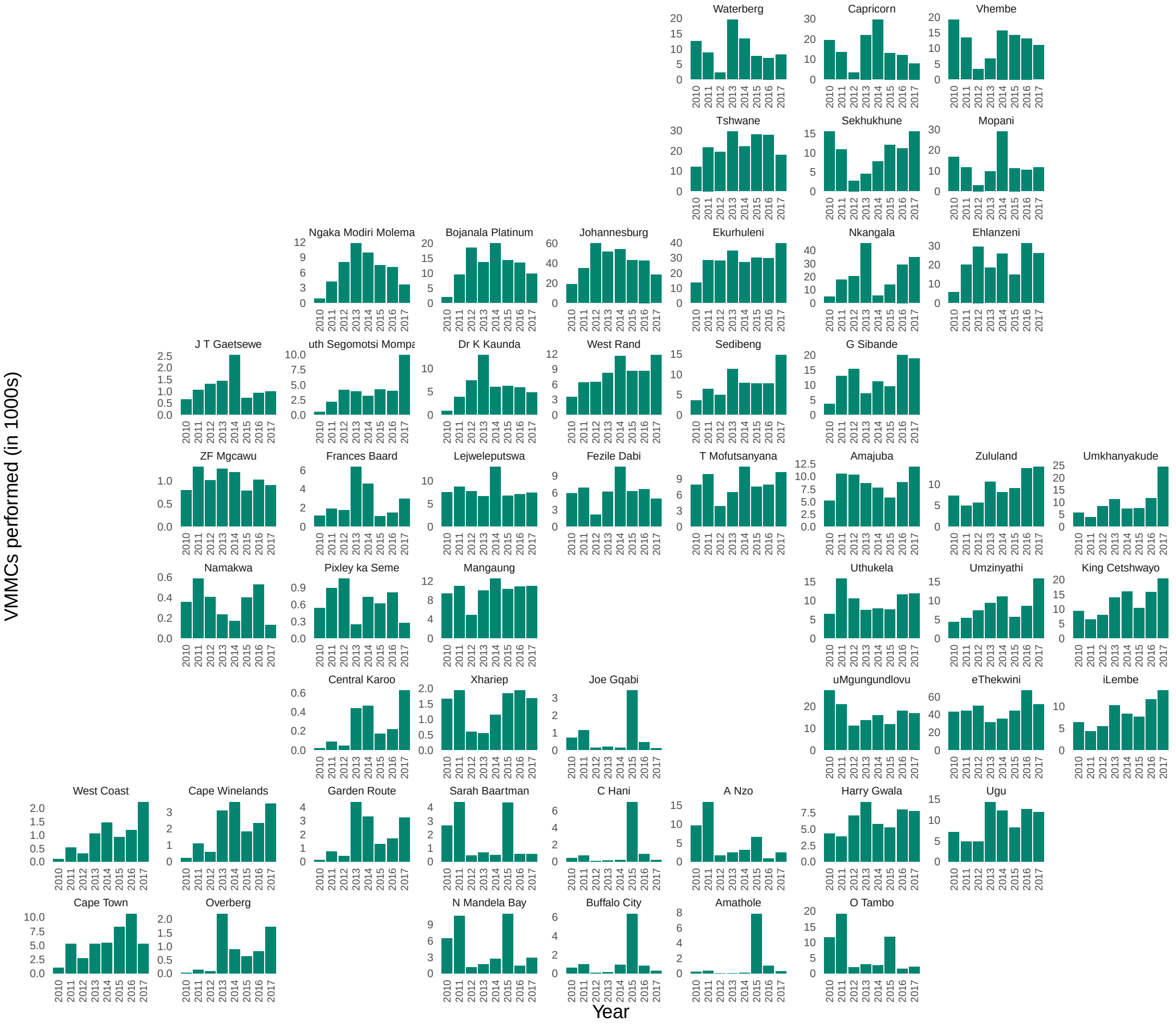

Supplementary Figure 4: Total number of VMMCs performed in each district between 2010 and 2017 reported to DMPPT2.

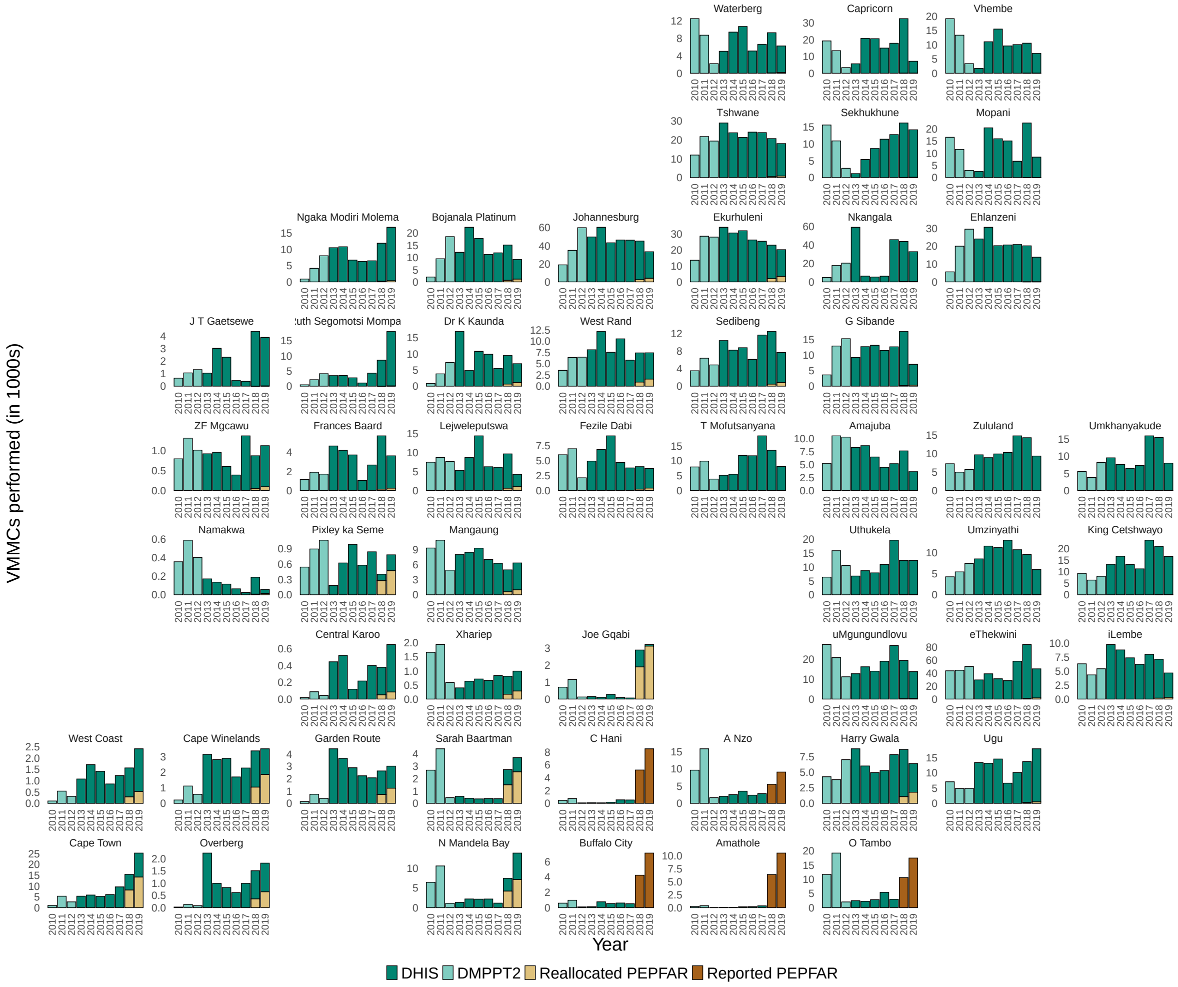

Supplementary Figure 5: Total number of VMMCs performed in each district between 2012 and 2019 used in the model. The primary source of information is from DHIS and is supplemented with data from DMPPT2 (2010-2012) and PEPFAR (in 2018-2019).

Supplementary Notes 2

Model fit - Coverage (MC)

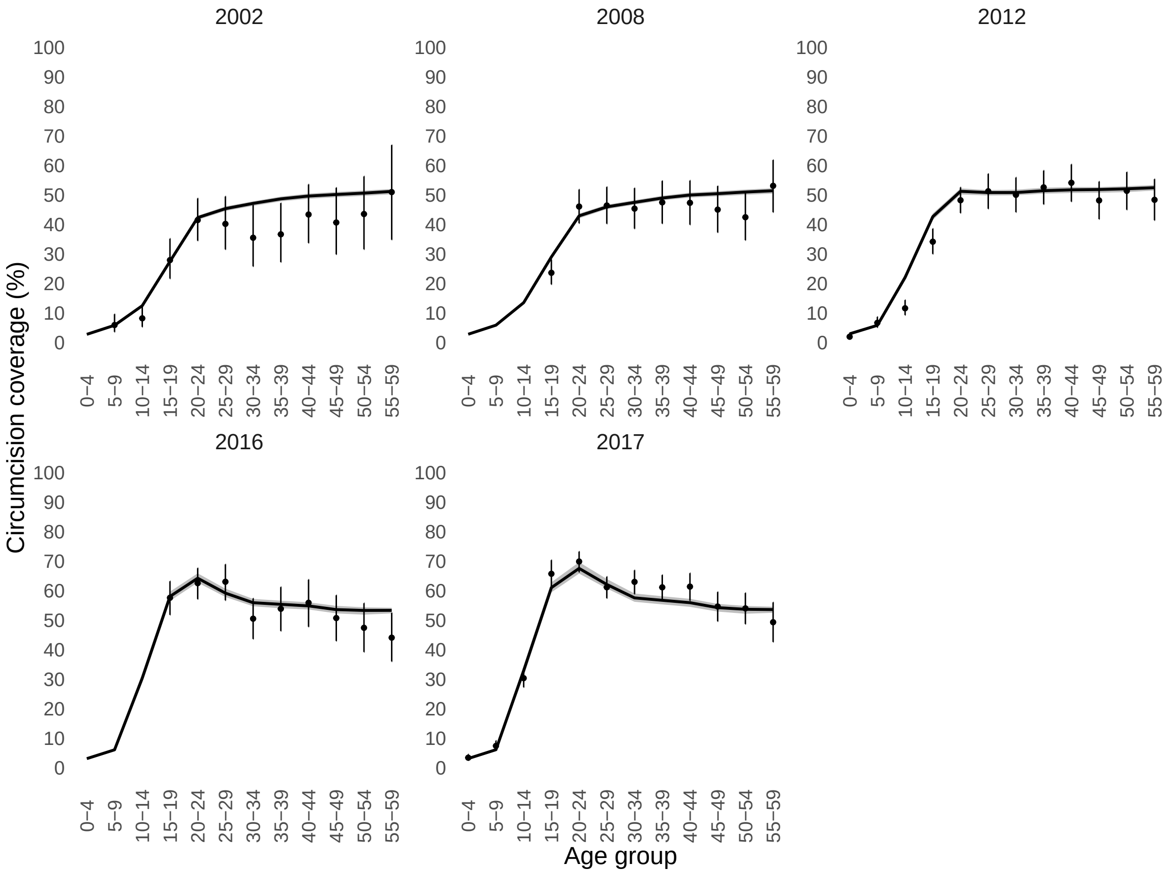

Supplementary Figure 6: National total MC coverage by 5-year age groups in 2002, 2008, 2012, 2016, and 2017. Lines denote the model estimated mean prevalence, with the shaded regions denoting the 95% CI. Black dots denote the direct survey estimates for MC coverage from the 2002, 2008, 2012 and 2017 SABSSM surveys and the 2016 DHS survey.

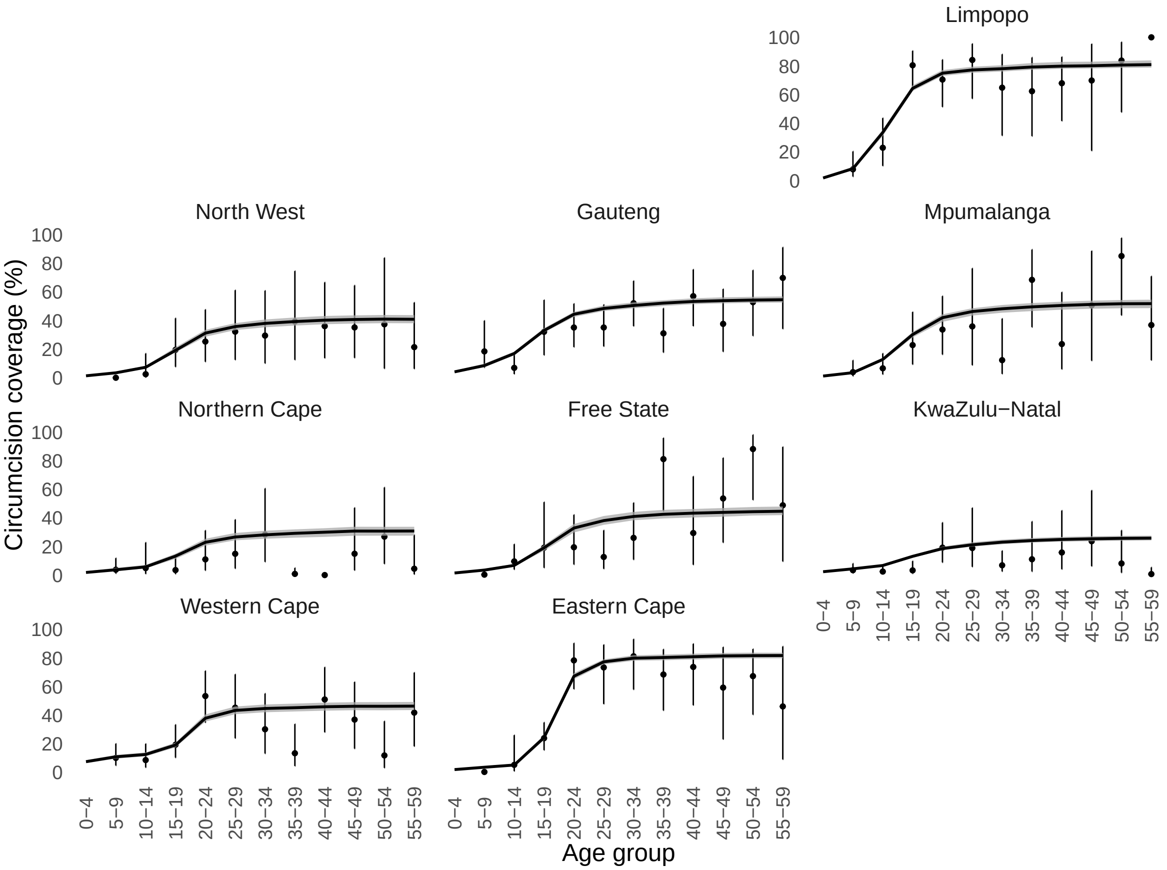

Supplementary Figure 7: Province-level MC coverage by 5-year age groups in 2002. Lines denote the estimated mean prevalence, with the shaded regions denoting the 95% CI. Black dots denote the sampled MC coverage from the 2002 SABSSM survey.

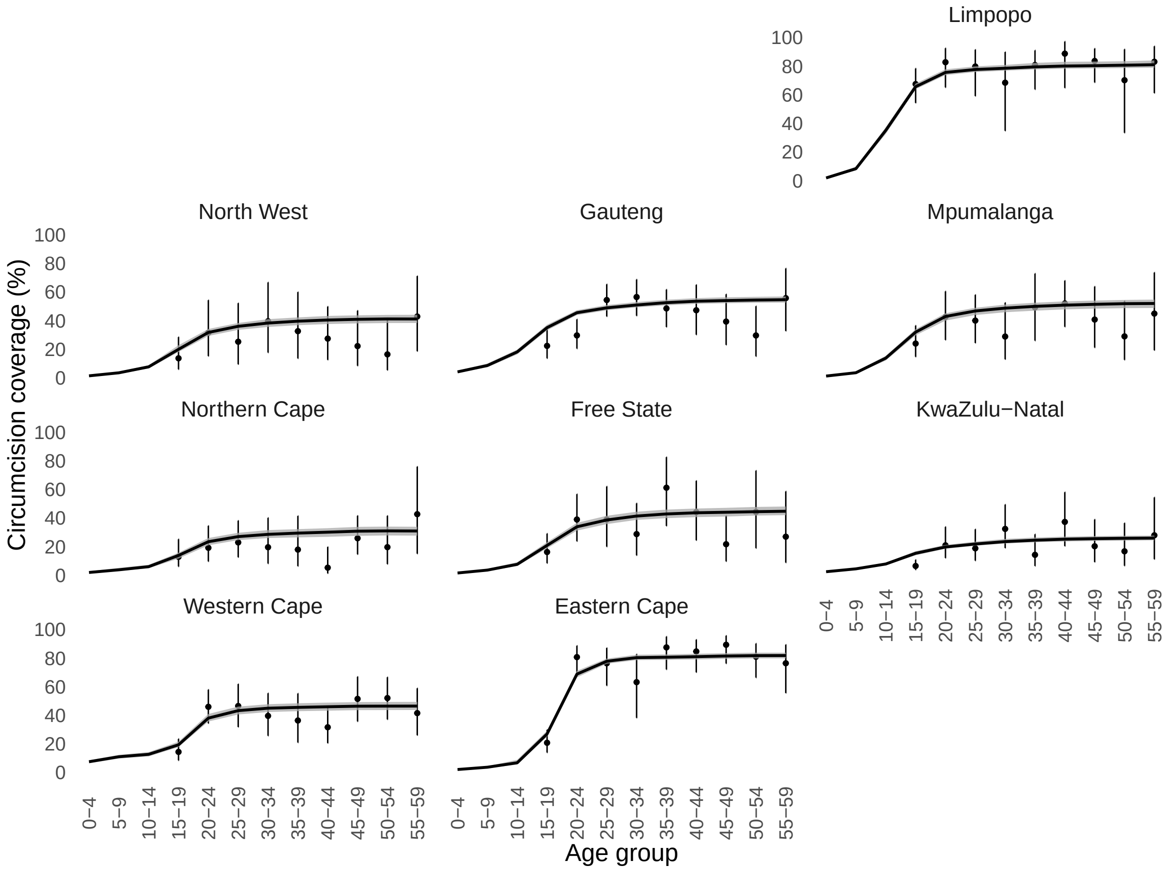

Supplementary Figure 8: Province-level MC coverage by 5-year age groups in 2008. Lines denote the estimated mean prevalence, with the shaded regions denoting the 95% CI. Black dots denote the sampled MC coverage from the 2008 SABSSM survey.

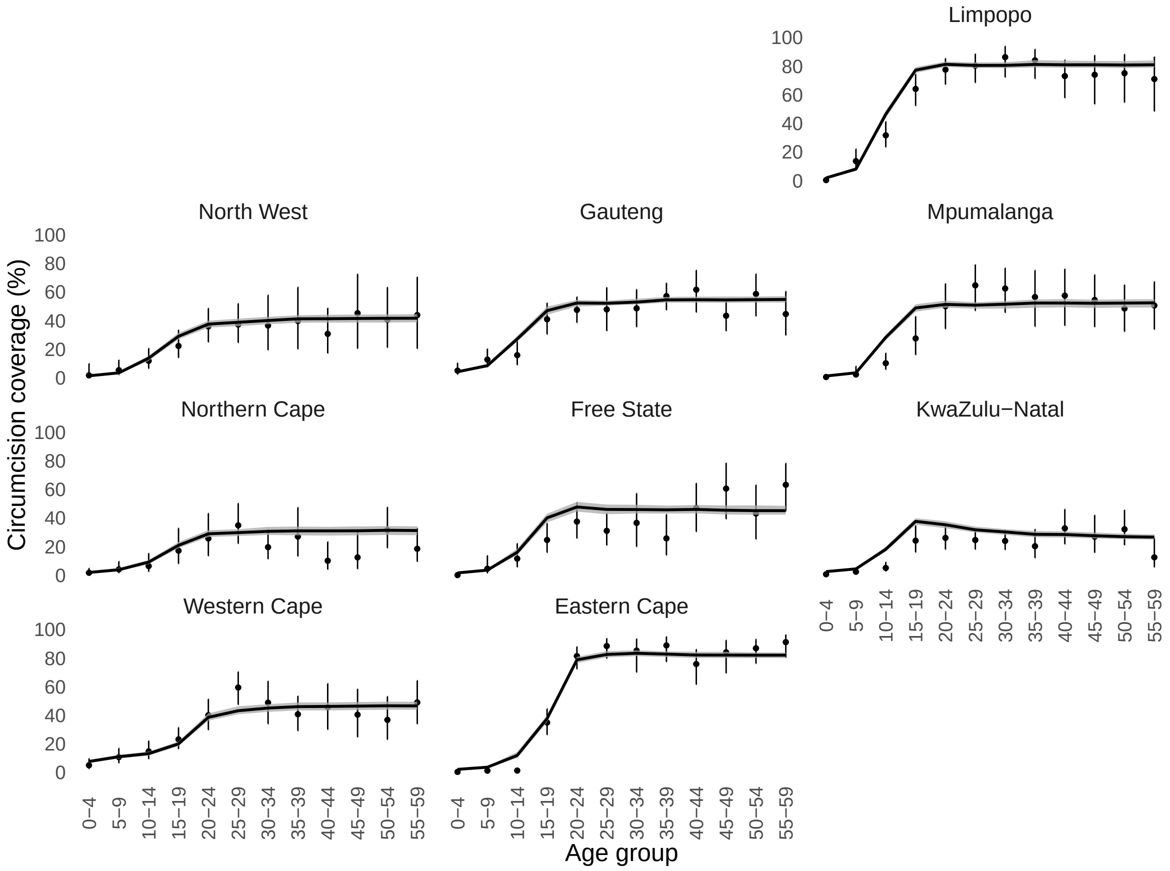

Supplementary Figure 9: Province-level MC coverage by 5-year age groups in 2012. Lines denote the estimated mean prevalence, with the shaded regions denoting the 95% CI. Black dots denote the sampled MC coverage from the 2012 SABSSM survey.

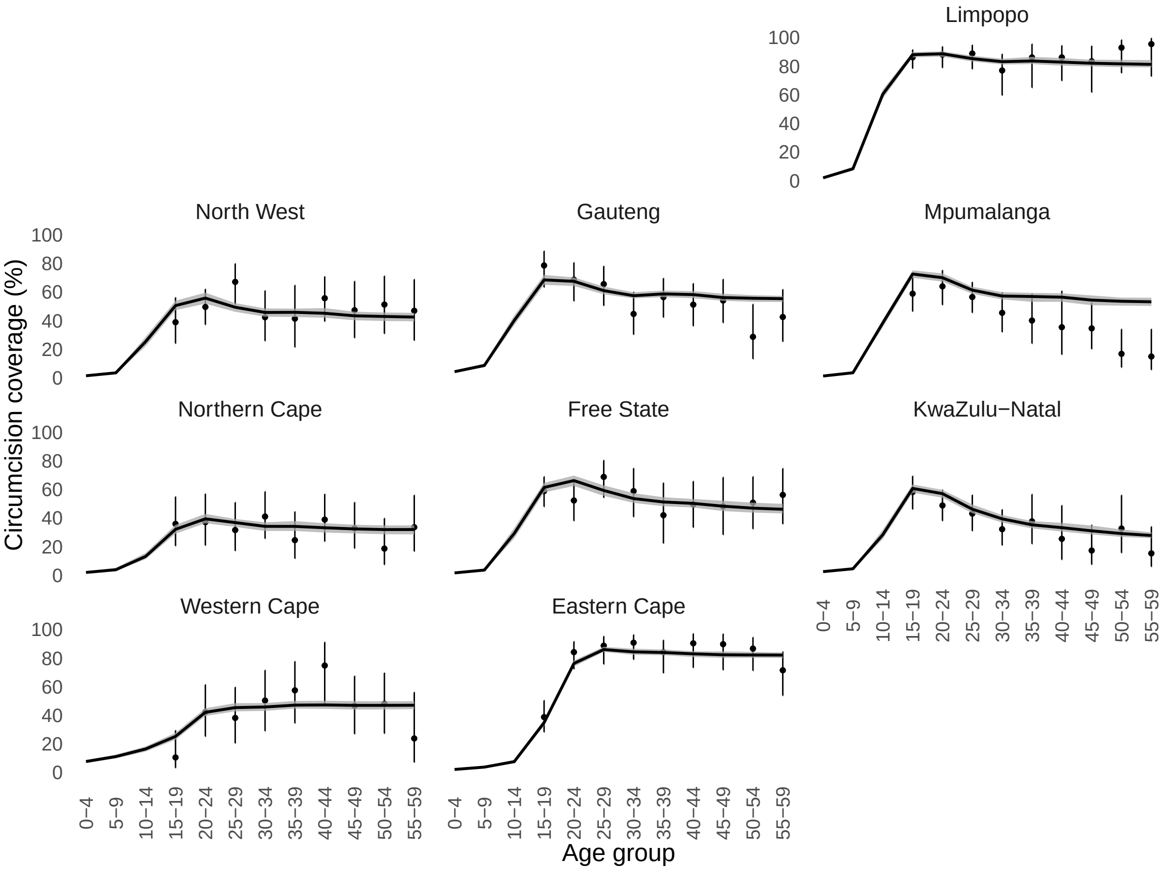

Supplementary Figure 10: Province-level MC coverage by 5-year age groups in 2016. Lines denote the estimated mean prevalence, with the shaded regions denoting the 95% CI. Black dots denote the sampled MC coverage from the 2016 DHS survey.

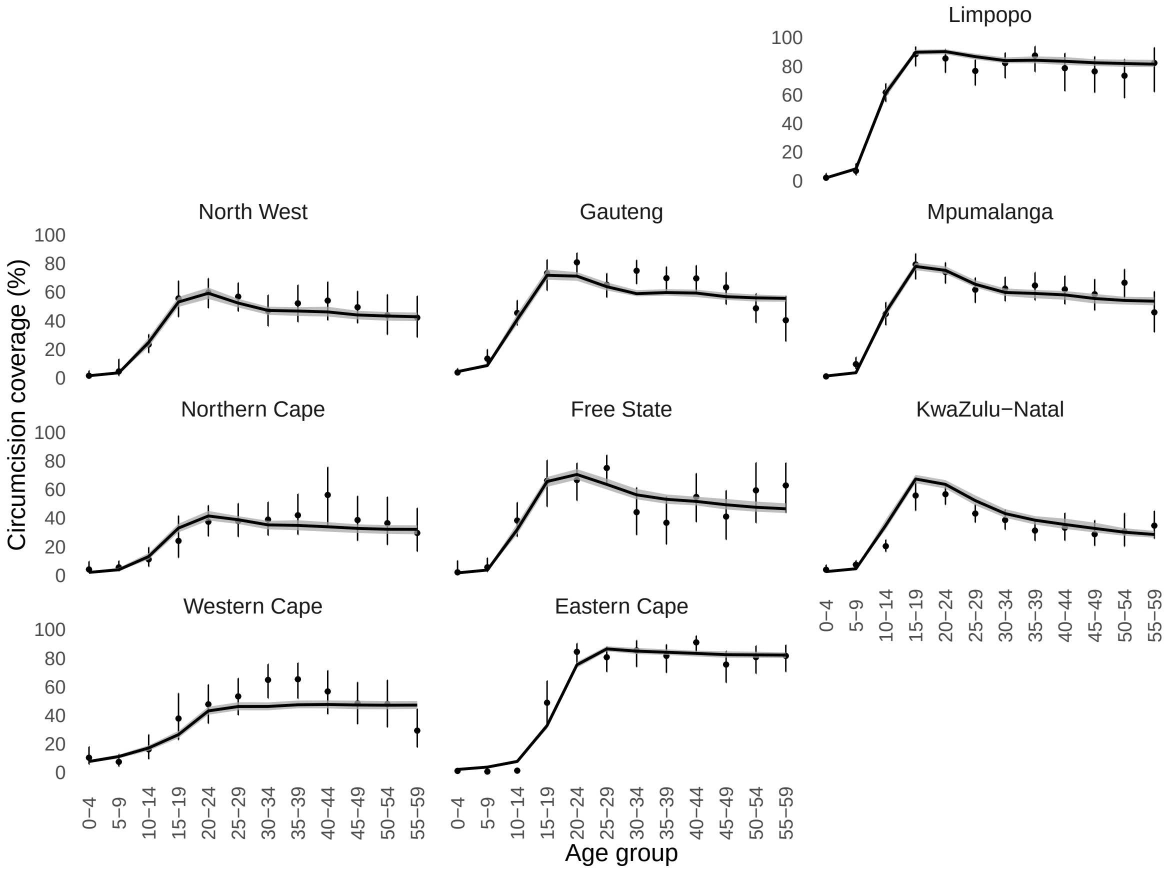

Supplementary Figure 11: Province-level MC coverage by 5-year age groups in 2017. Lines denote the estimated mean prevalence, with the shaded regions denoting the 95% CI. Black dots denote the sampled MC coverage from the 2017 SABSSM survey.

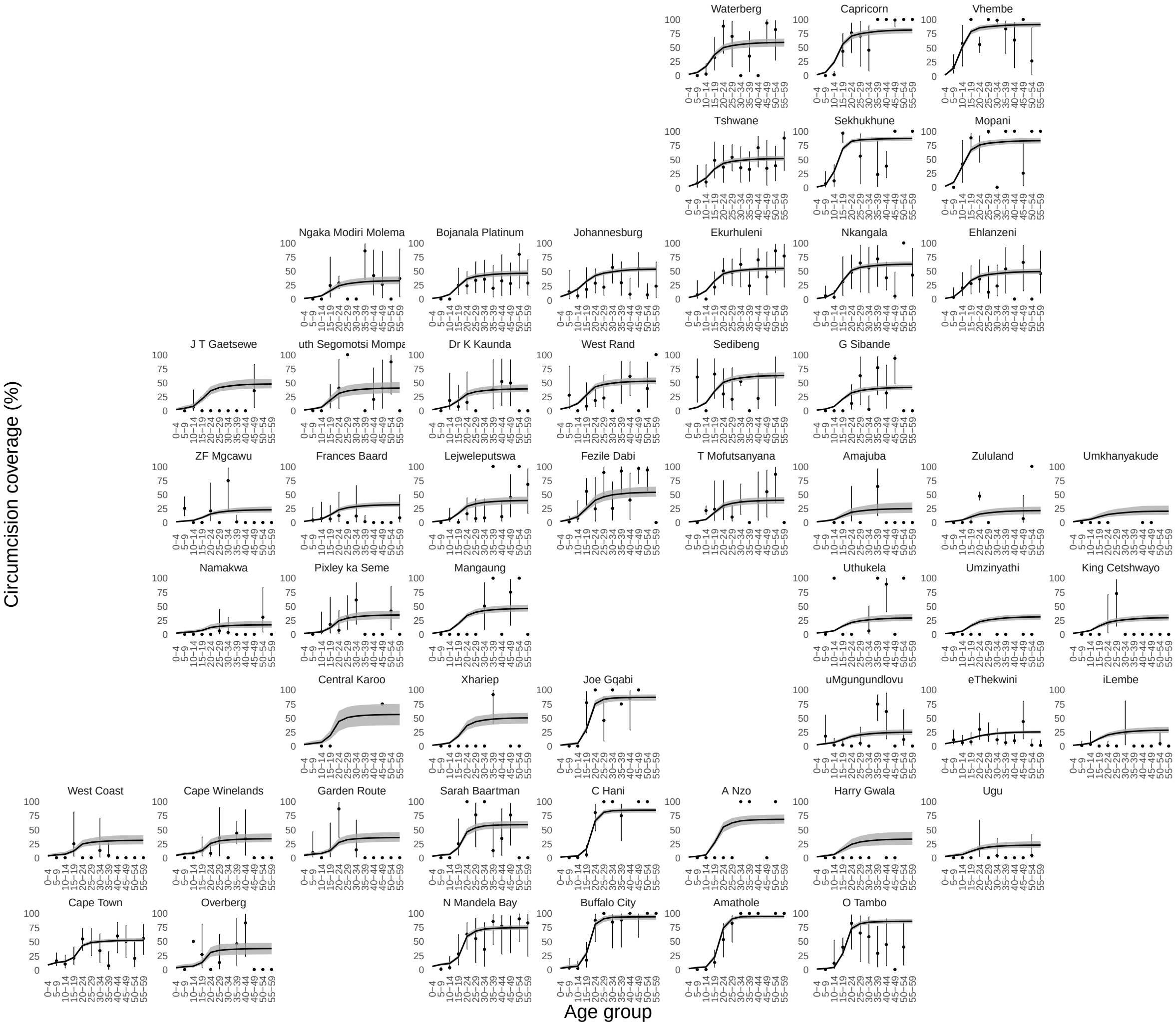

Supplementary Figure 12: District-level MC coverage by 5-year age groups in 2002 at a district level. Lines denote the estimated mean prevalence, with the shaded regions denoting the 95% CI. Black dots denote the sampled MC coverage from the 2002 SABSSM survey.

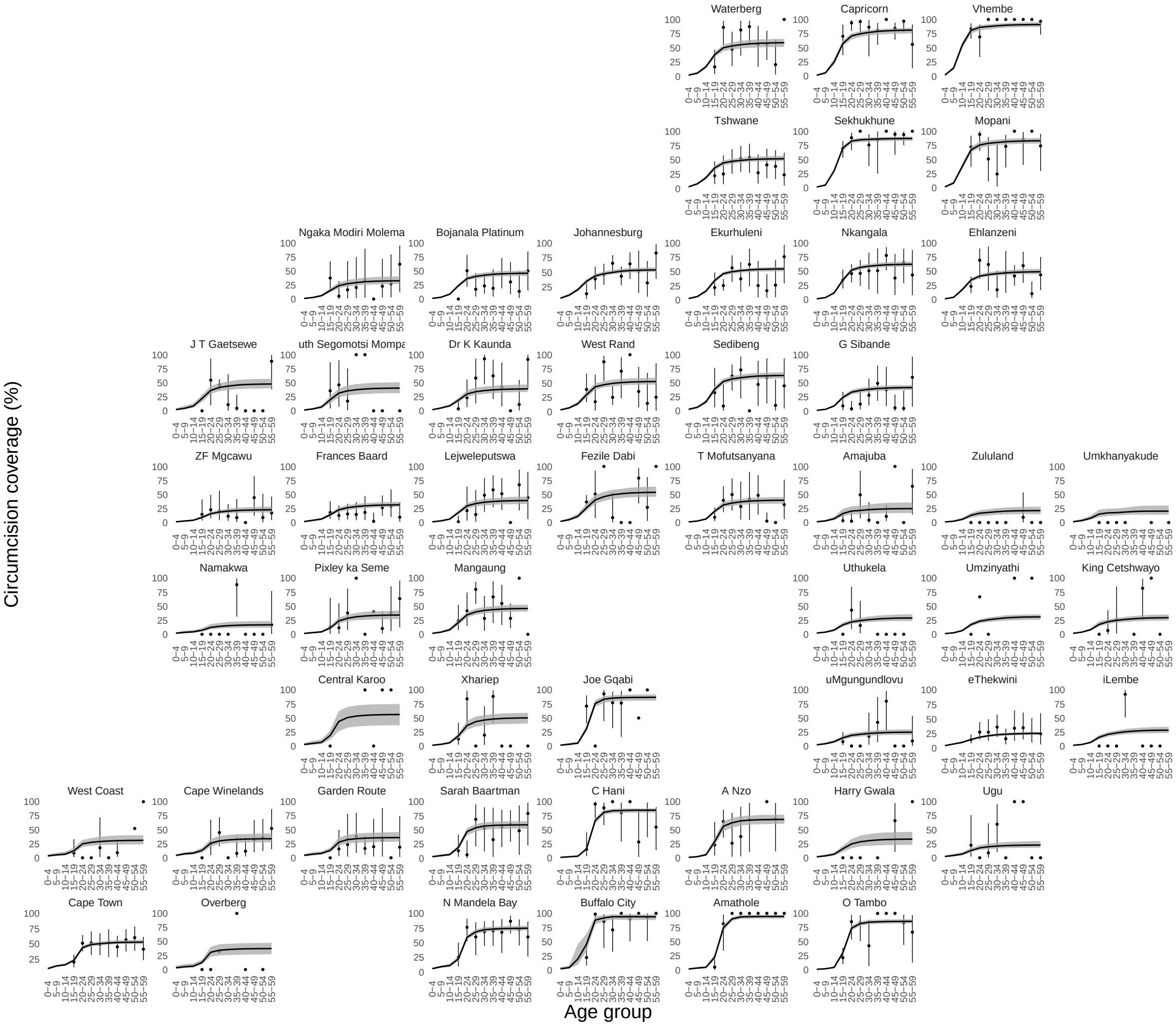

Supplementary Figure 13: District-level MC coverage by 5-year age groups in 2008. Lines denote the estimated mean prevalence, with the shaded regions denoting the 95% CI. Black dots denote the sampled MC coverage from the 2008 SABSSM survey.

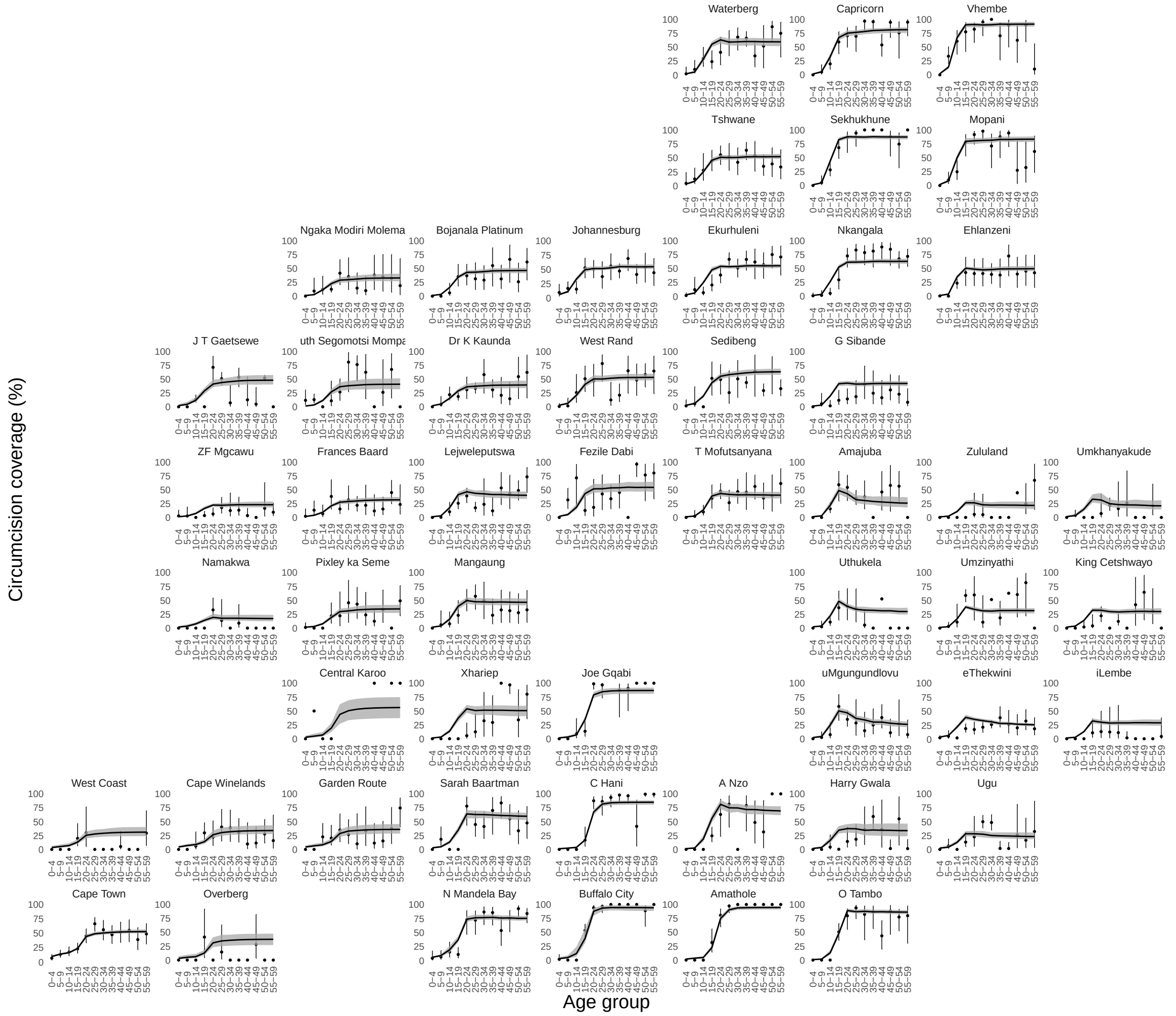

Supplementary Figure 14: District-level MC coverage by 5-year age groups in 2012. Lines denote the estimated mean prevalence, with the shaded regions denoting the 95% CI. Black dots denote the sampled MC coverage from the 2012 SABSSM survey.

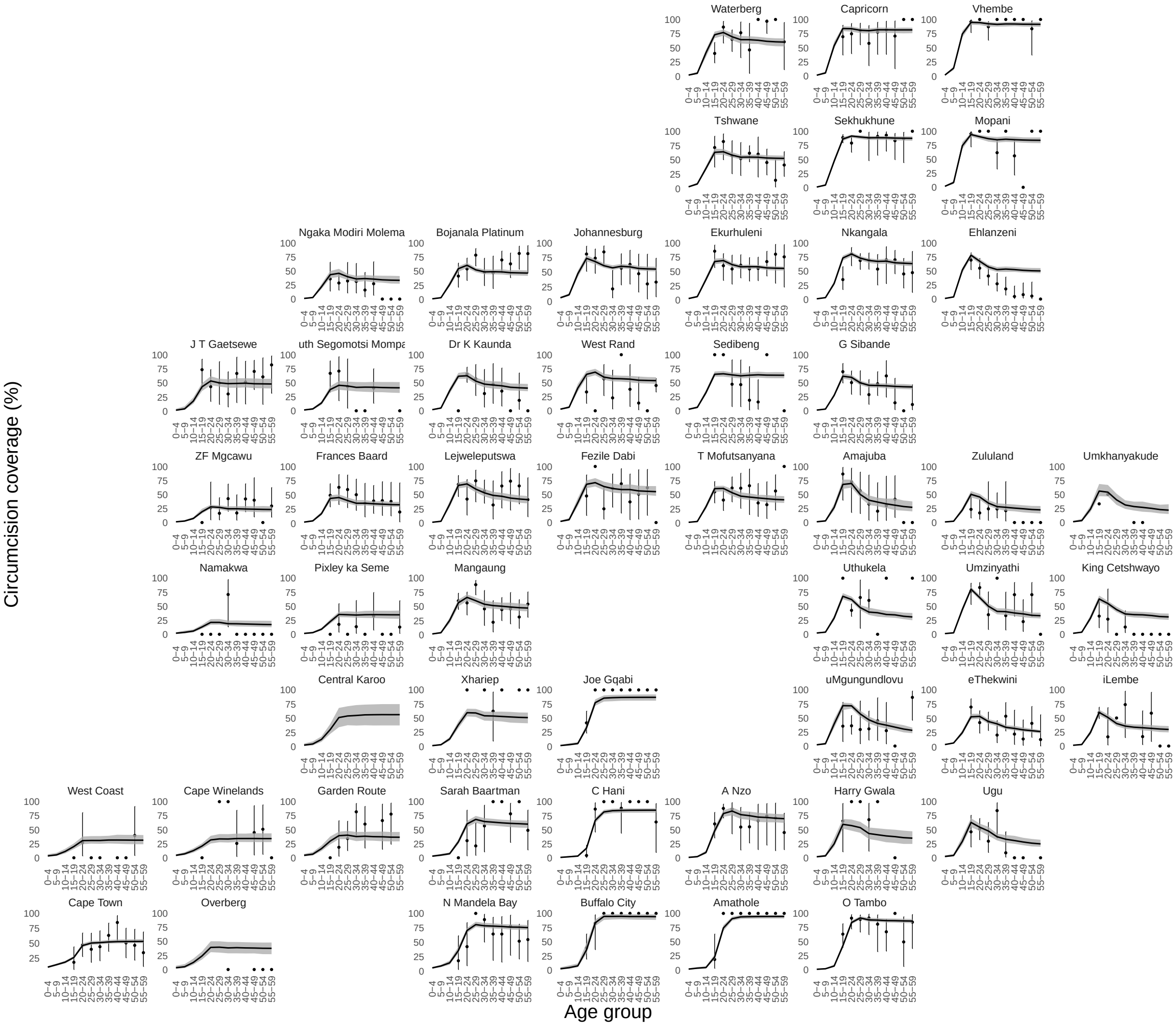

Supplementary Figure 15: District-level MC coverage by 5-year age groups in 2016. Lines denote the estimated mean prevalence, with the shaded regions denoting the 95% CI. Black dots denote the sampled MC coverage from the 2016 DHS survey.

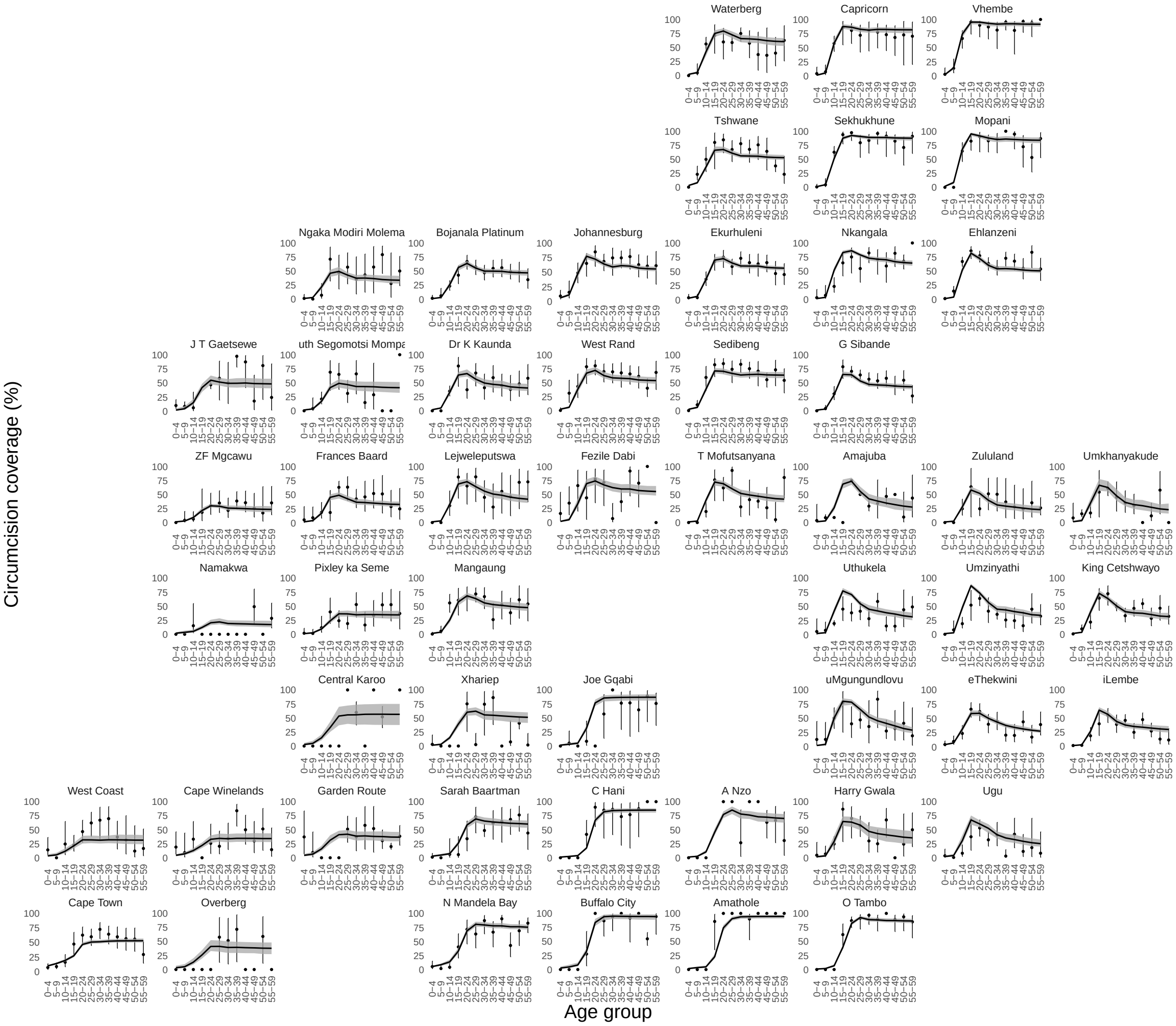

Supplementary Figure 16: District-level MC coverage by 5-year age groups in 2017. Lines denote the estimated mean prevalence, with the shaded regions denoting the 95% CI. Black dots denote the sampled MC coverage from the 2017 SABSSM survey.

Model fit - Coverage of medical male circumcision (MMC-nT)

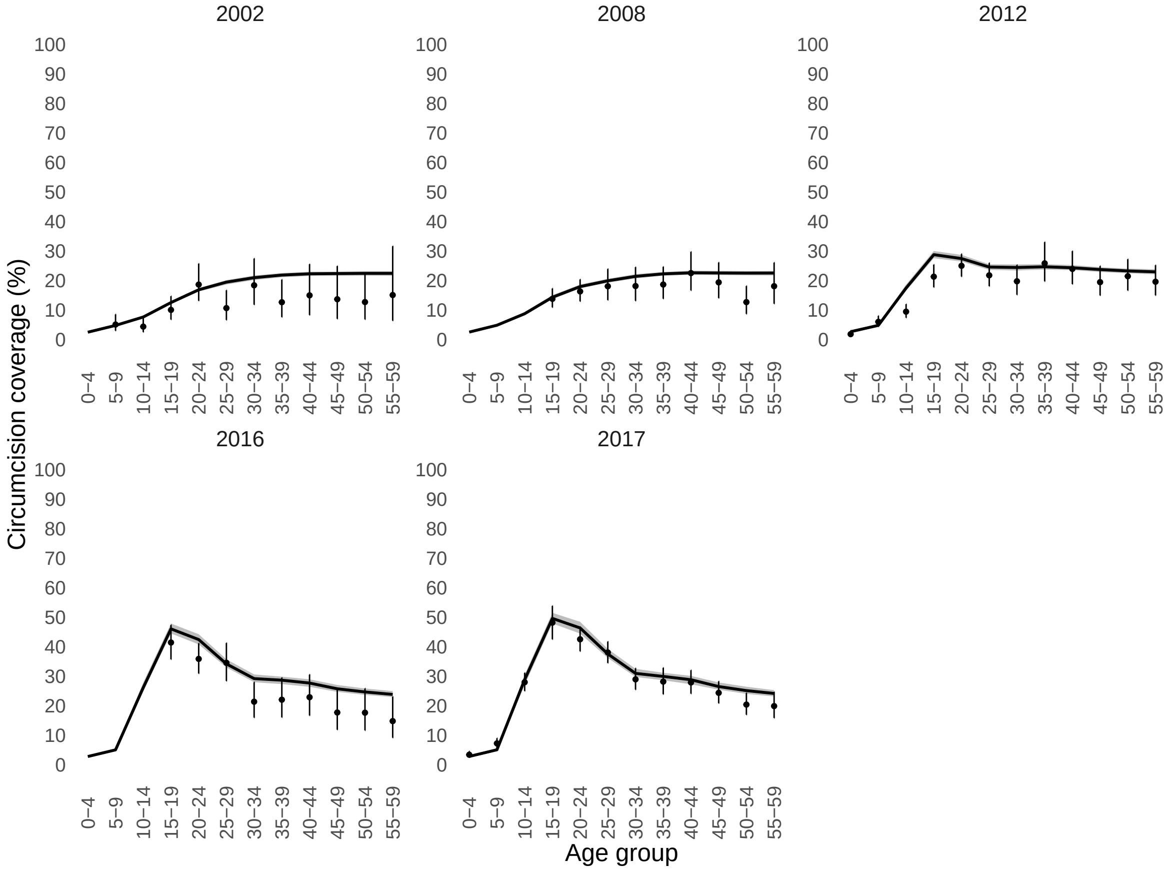

Supplementary Figure 17: National MMC-nT coverage by 5-year age groups in 2002, 2008, 2012, 2016 and 2017. Lines denote the estimated mean prevalence, with the shaded regions denoting the 95% CI. Black dots denote the sampled MMC-nT coverage from the 2002, 2008, 2012 and 2017 SABSSM survey as well as the 2016 DHS survey.

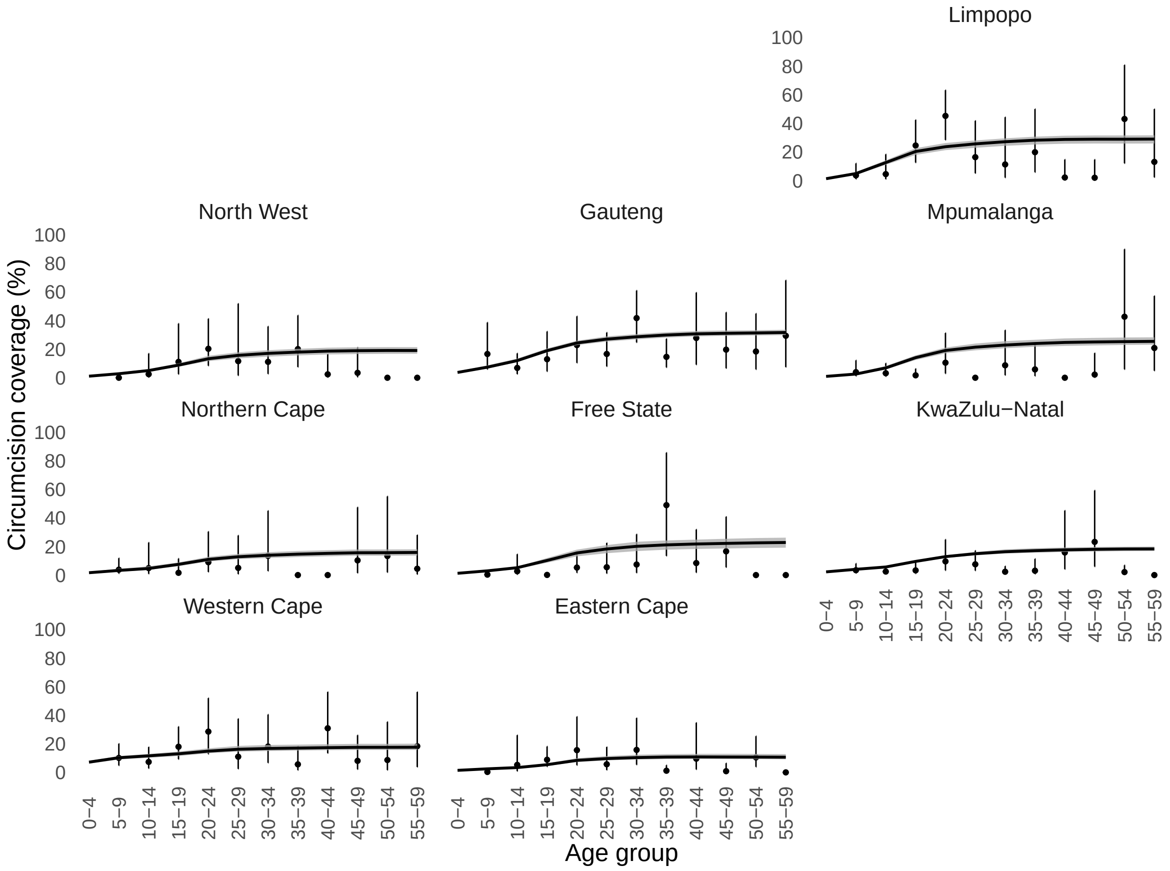

Supplementary Figure 18: Province-level MMC-nT coverage by 5-year age groups in 2002. Lines denote the estimated mean prevalence, with the shaded regions denoting the 95% CI. Black dots denote the sampled MMC-nT coverage from the 2002 SABSSM survey.

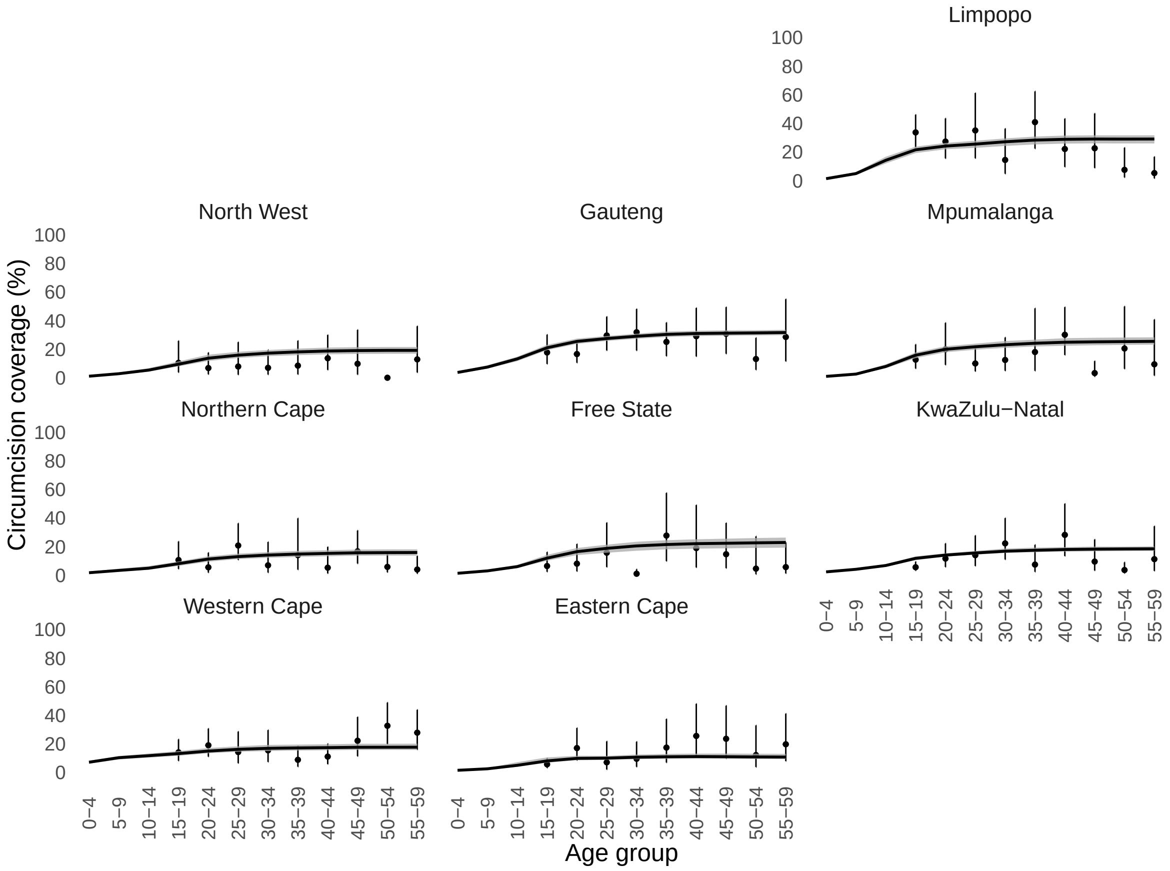

Supplementary Figure 19: Province-level MMC-nT coverage by 5-year age groups in 2008. Lines denote the estimated mean prevalence, with the shaded regions denoting the 95% CI. Black dots denote the sampled MMC-nT coverage from the 2008 SABSSM survey.

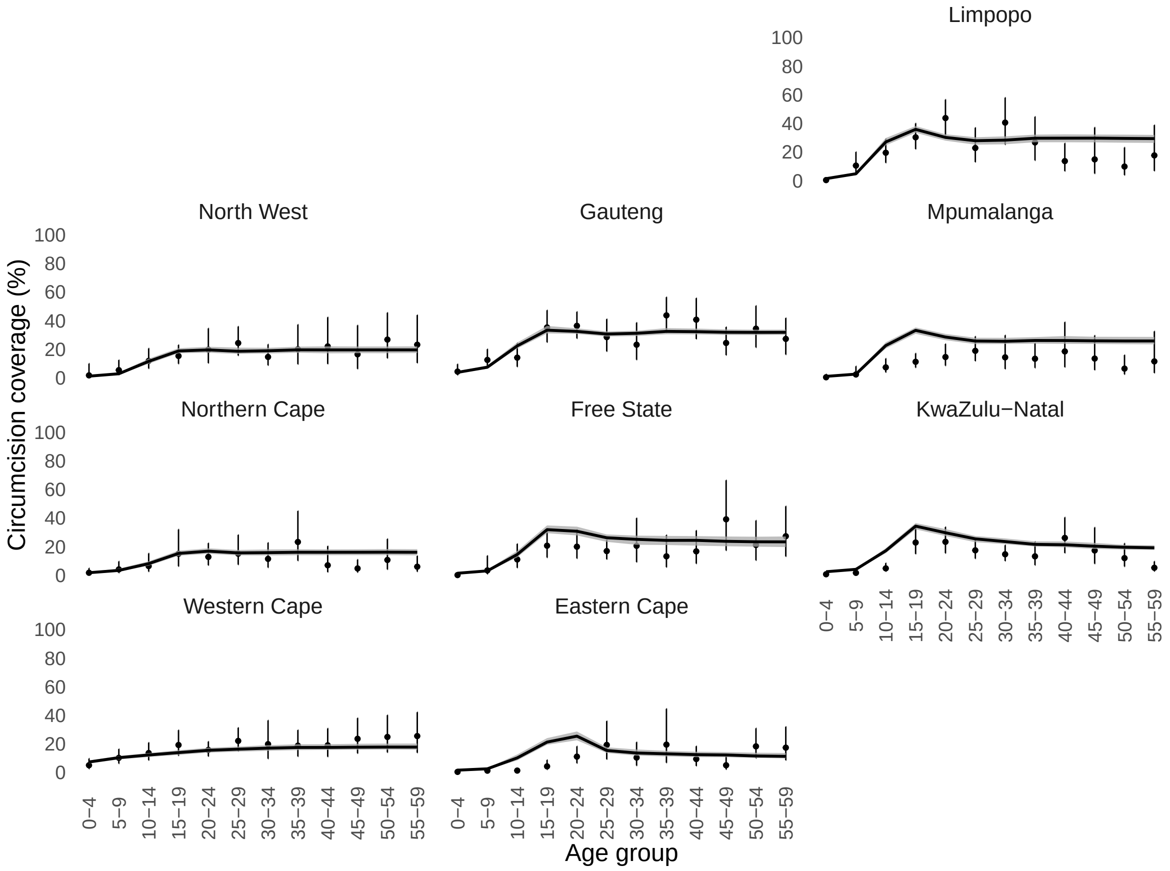

Supplementary Figure 20: Province-level MMC-nT coverage by 5-year age groups in 2012. Lines denote the estimated mean prevalence, with the shaded regions denoting the 95% CI. Black dots denote the sampled MMC-nT coverage from the 2012 SABSSM survey.

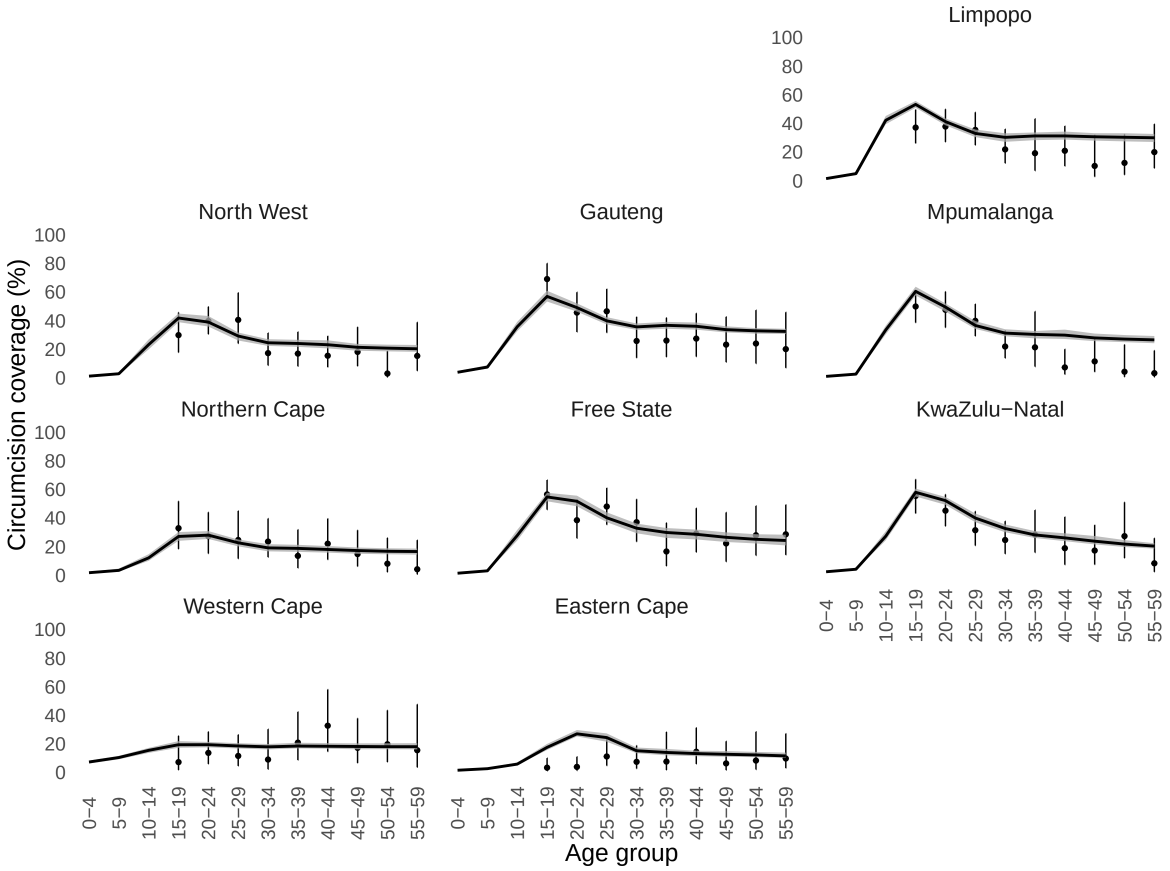

Supplementary Figure 21: Province-level MMC-nT coverage by 5-year age groups in 2016. Lines denote the estimated mean prevalence, with the shaded regions denoting the 95% CI. Black dots denote the sampled MMC-nT coverage from the 2016 DHS survey.

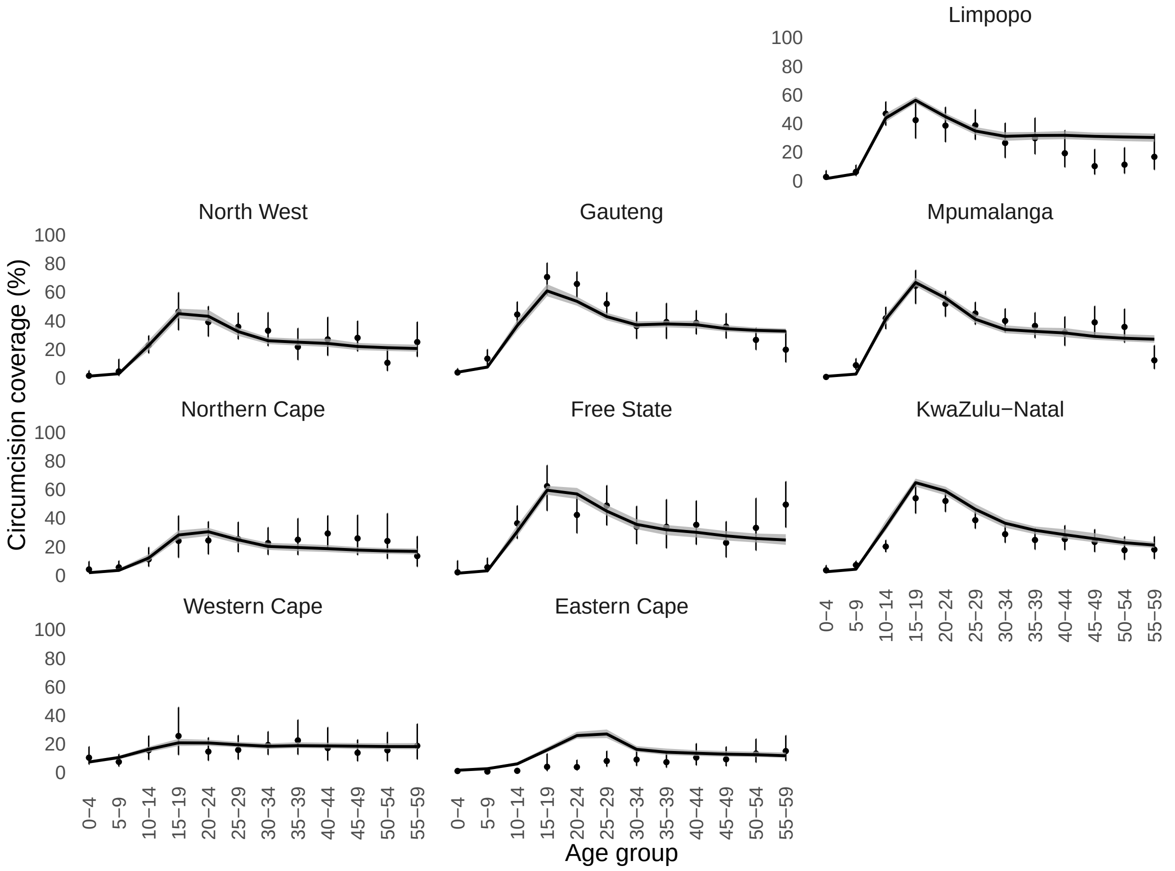

Supplementary Figure 22: Province-level MMC-nT coverage by 5-year age groups in 2017. Lines denote the estimated mean prevalence, with the shaded regions denoting the 95% CI. Black dots denote the sampled MMC-nT coverage from the 2017 SABSSM survey.

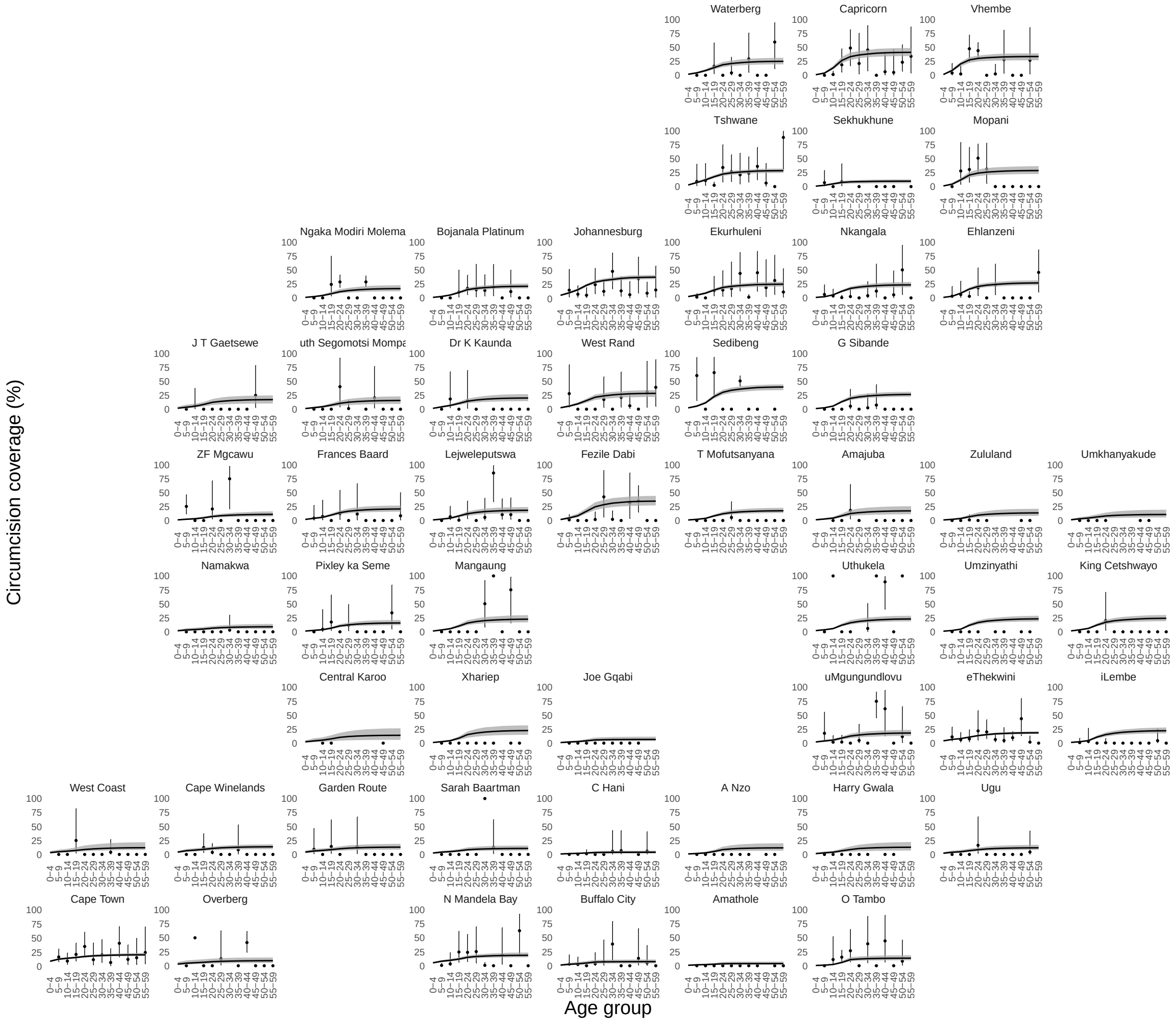

Supplementary Figure 23: District-level Estimated MMC-nT coverage by 5-year age groups in 2002. Lines denote the estimated mean prevalence, with the shaded regions denoting the 95% CI. Black dots denote the sampled MMC-nT coverage from the 2002 SABSSM survey.

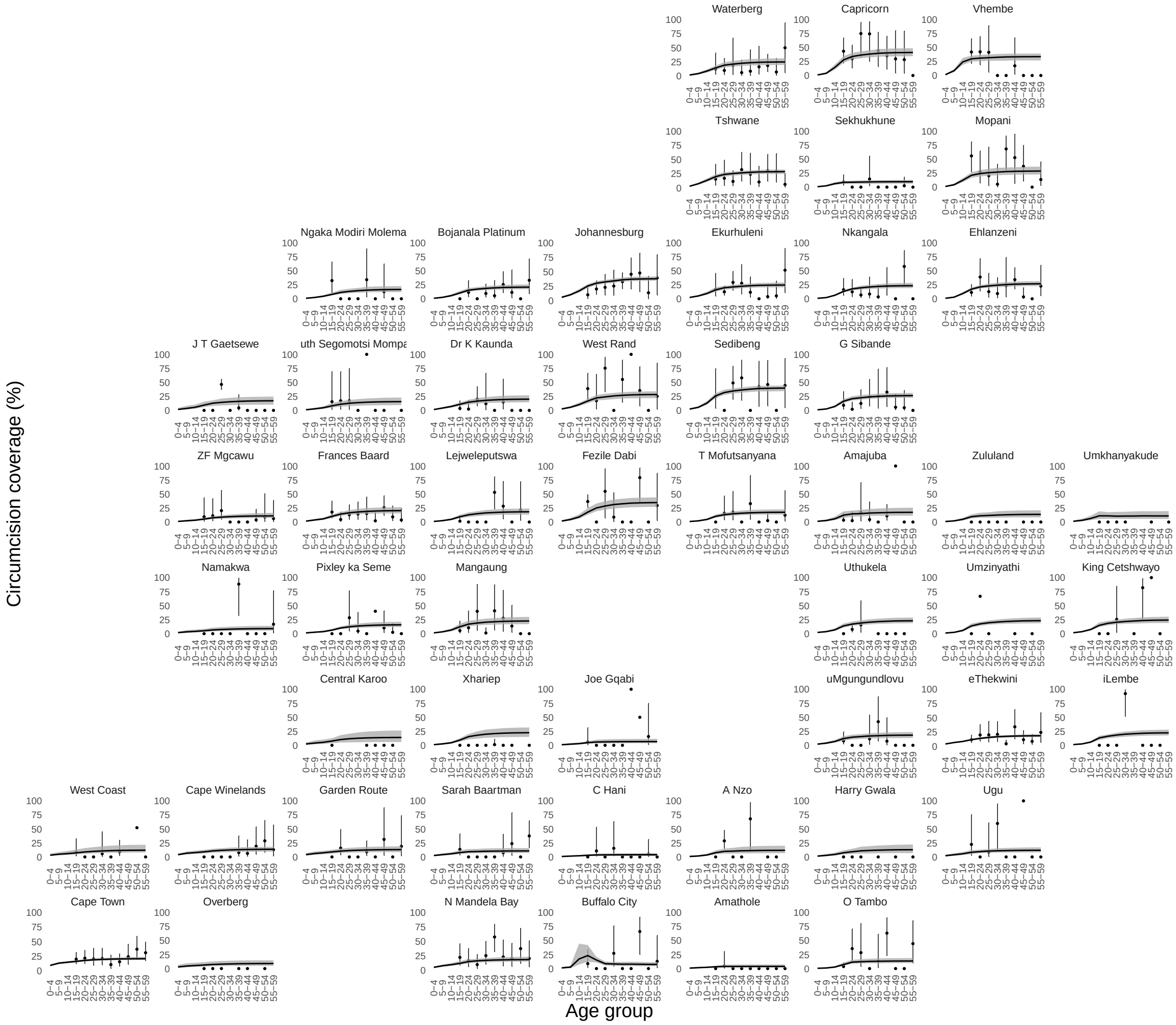

Supplementary Figure 24: District-level MMC-nT coverage by 5-year age groups in 2008. Lines denote the estimated mean prevalence, with the shaded regions denoting the 95% CI. Black dots denote the sampled MMC-nT coverage from the 2008 SABSSM survey.

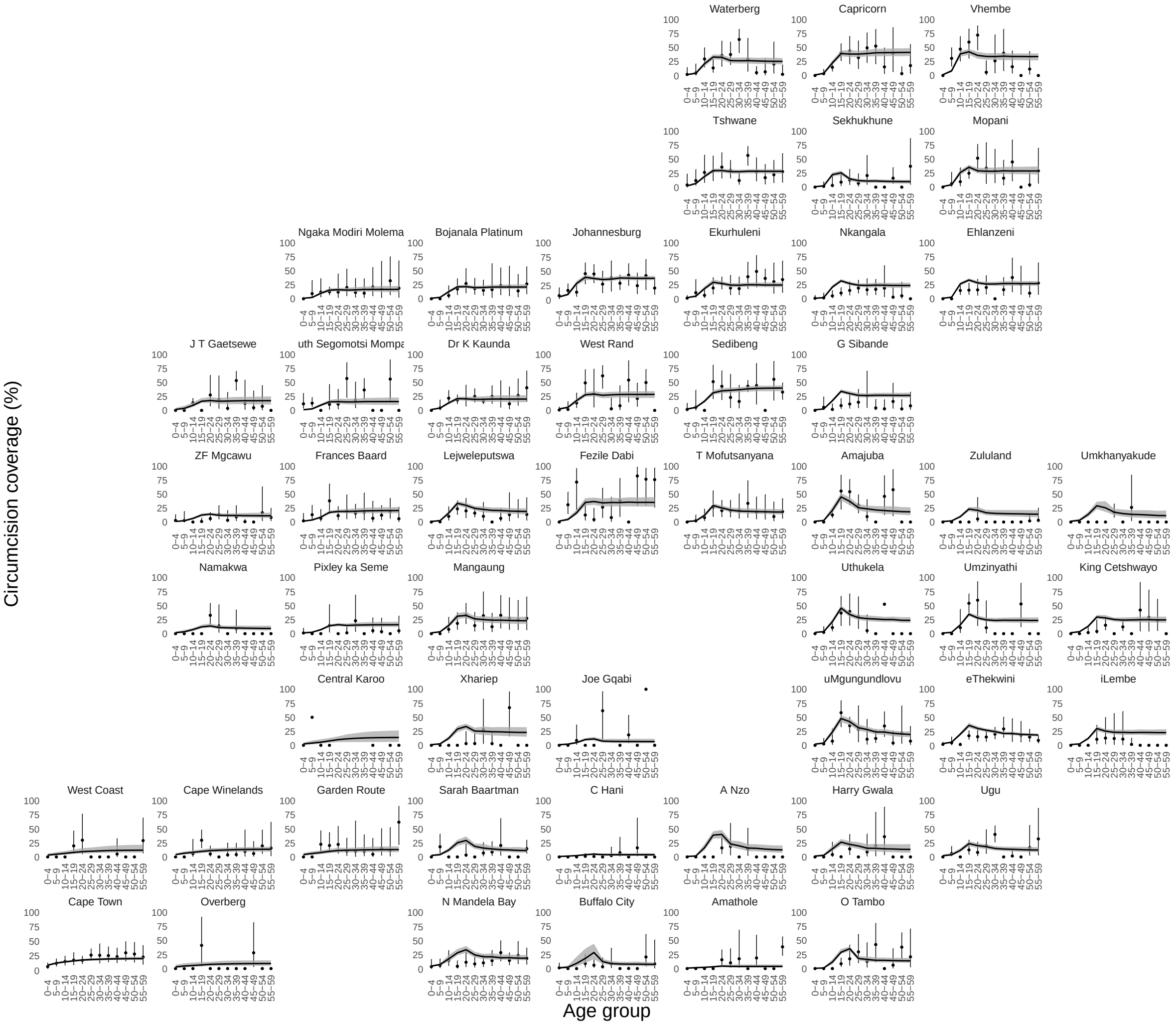

Supplementary Figure 25: District-level MMC-nT coverage by 5-year age groups in 2012. Lines denote the estimated mean prevalence, with the shaded regions denoting the 95% CI. Black dots denote the sampled MMC-nT coverage from the 2012 SABSSM survey.

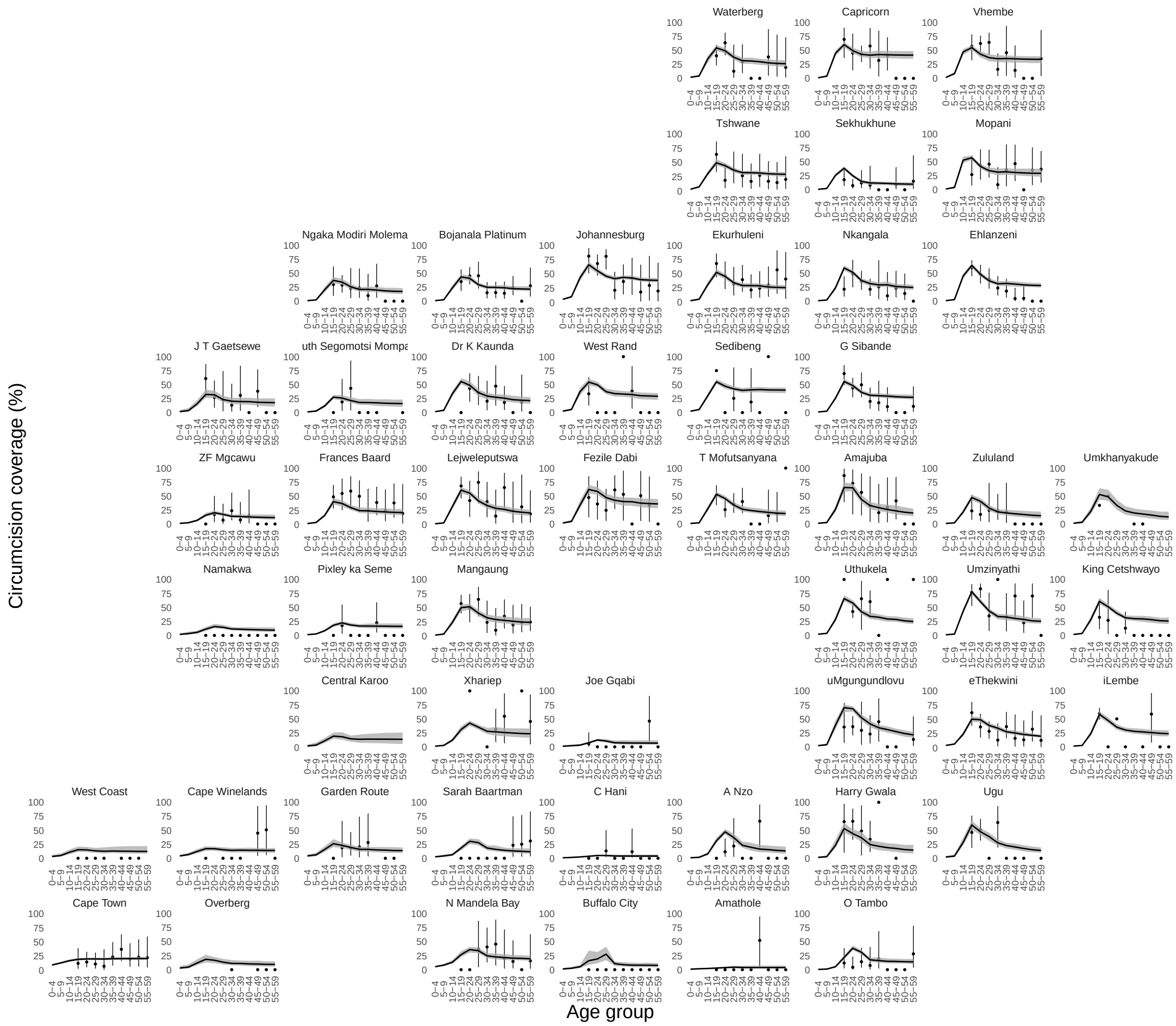

Supplementary Figure 26: District-level MMC-nT coverage by 5-year age groups in 2016. Lines denote the estimated mean prevalence, with the shaded regions denoting the 95% CI. Black dots denote the sampled MMC-nT coverage from the 2016 DHS survey.

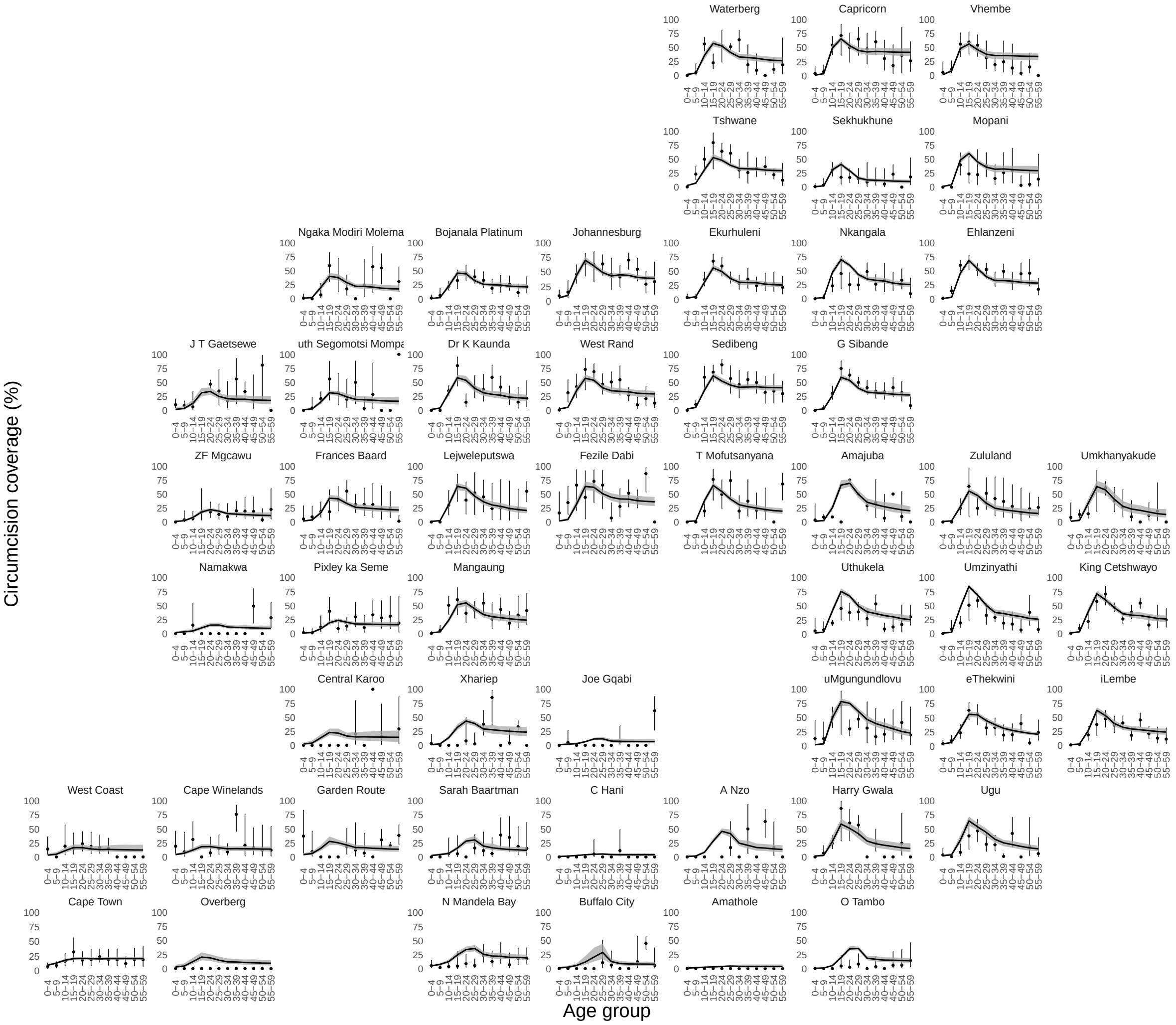

Supplementary Figure 27: District-level MMC-nT coverage by 5-year age groups in 2017. Lines denote the estimated mean prevalence, with the shaded regions denoting the 95% CI. Black dots denote the sampled MMC-nT coverage from the 2017 SABSSM survey.

Model fit - Coverage of traditional male circumcision (TMIC)

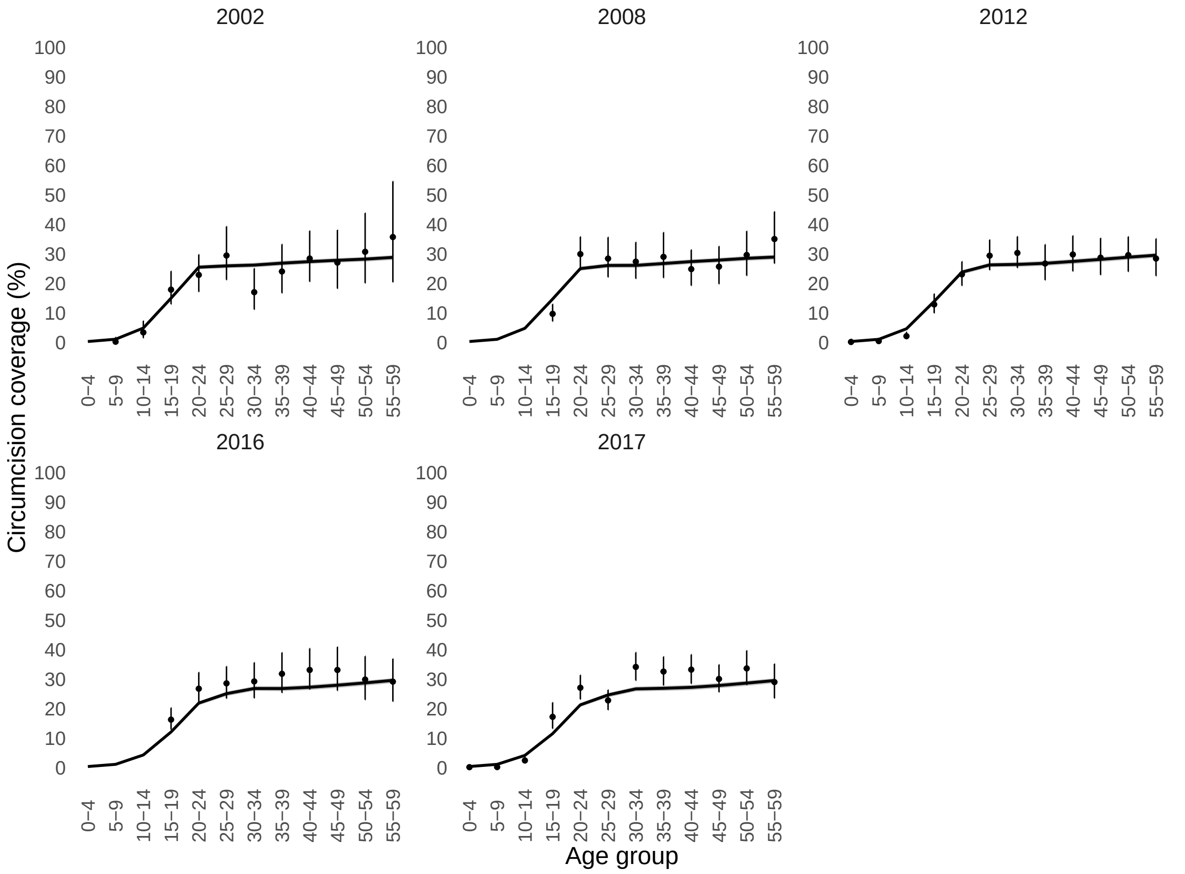

Supplementary Figure 28: Natonial TMIC coverage by 5-year age groups in 2002, 2008, 2012, 2016 and 2017. Lines denote the estimated mean prevalence, with the shaded regions denoting the 95% CI. Black dots denote the sampled TMIC coverage from the 2002, 2008, 2012, and 2017 SABSSM surveys and the 2016 DHS survey.

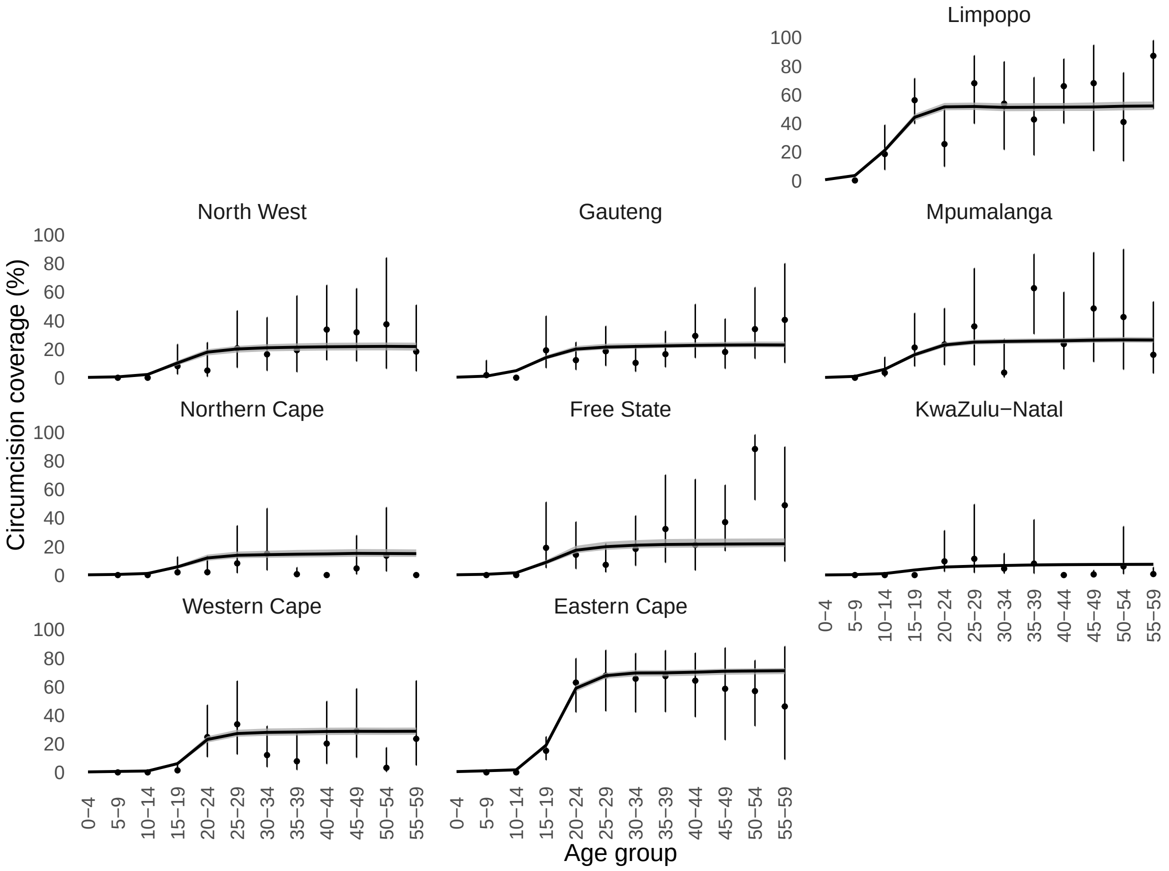

Supplementary Figure 29: Province-level TMIC coverage by 5-year age groups in 2002. Lines denote the estimated mean prevalence, with the shaded regions denoting the 95% CI. Black dots denote the sampled TMIC coverage from the 2002 SABSSM survey.

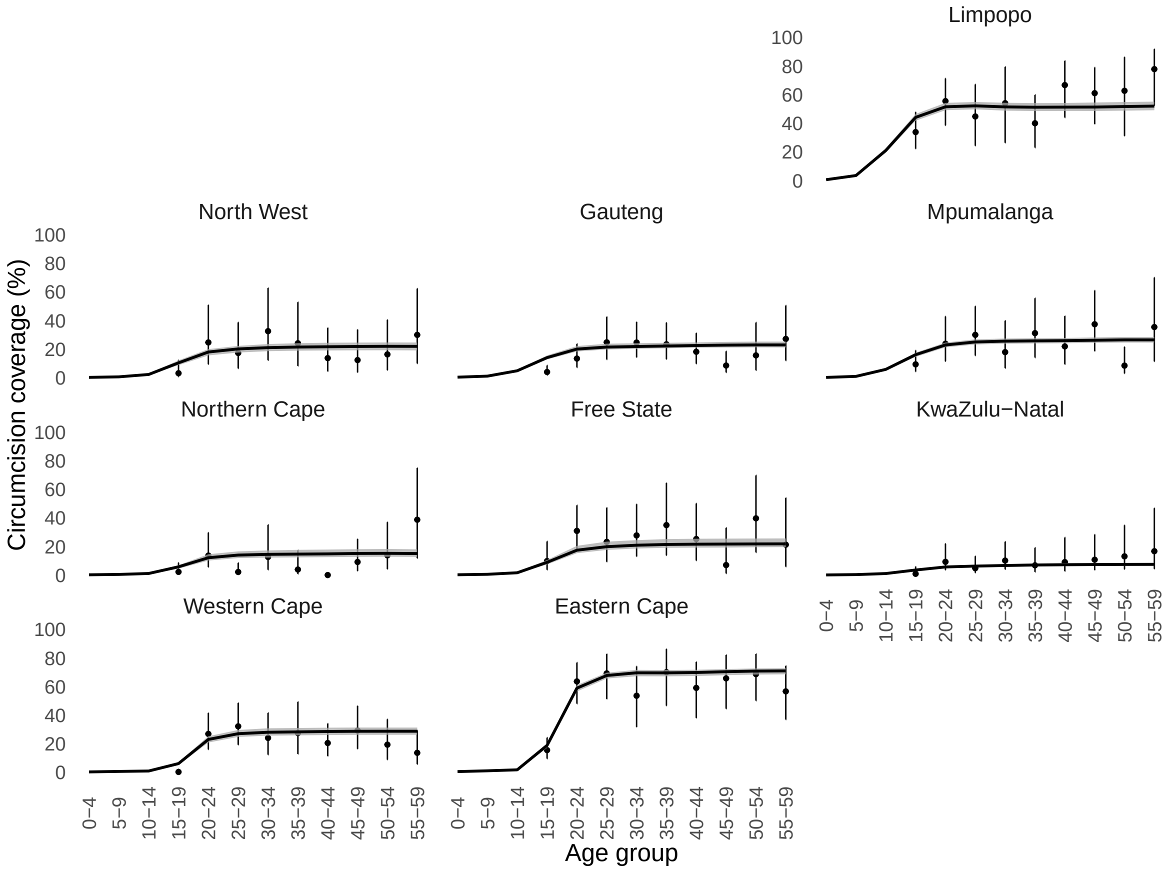

Supplementary Figure 30: Province-level TMIC coverage by 5-year age groups in 2008. Lines denote the estimated mean prevalence, with the shaded regions denoting the 95% CI. Black dots denote the sampled TMIC coverage from the 2008 SABSSM survey.

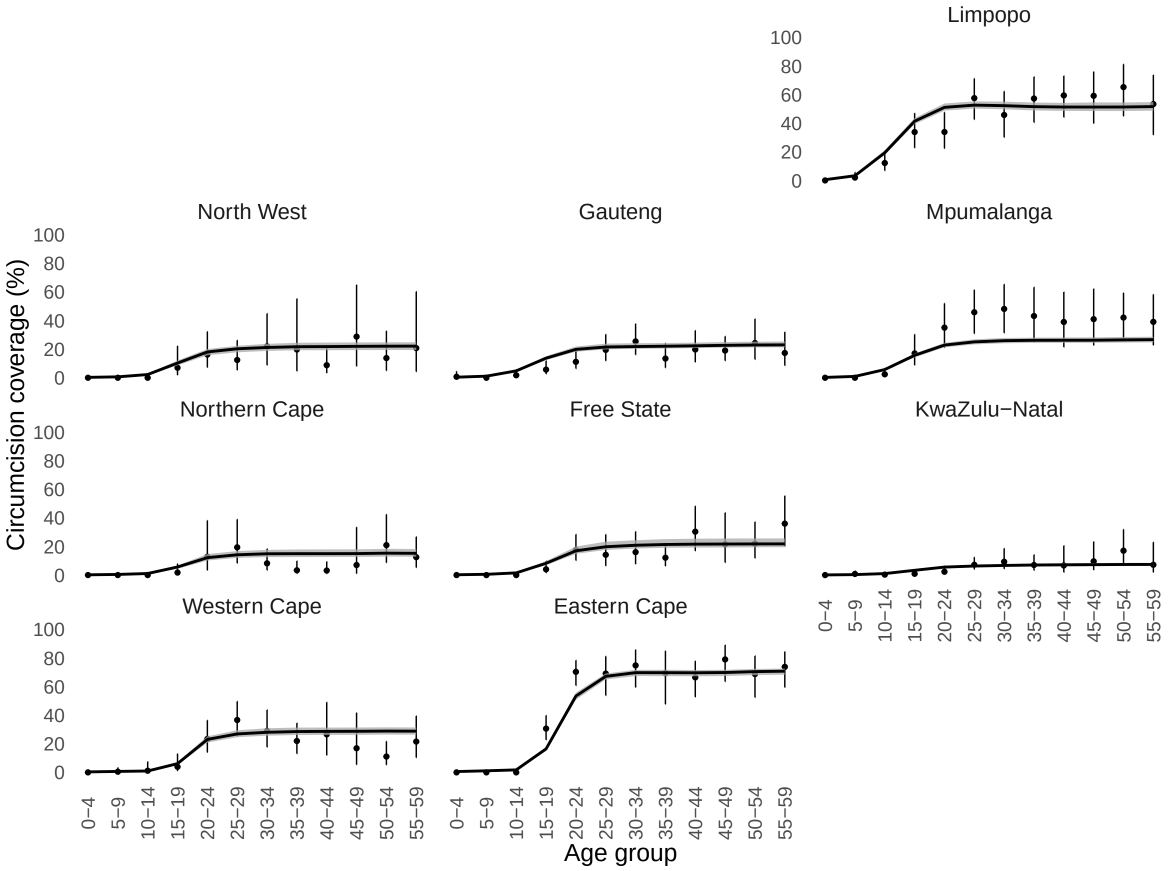

Supplementary Figure 31: Province-level TMIC coverage by 5-year age groups in 2012. Lines denote the estimated mean prevalence, with the shaded regions denoting the 95% CI. Black dots denote the sampled TMIC coverage from the 2012 SABSSM survey.

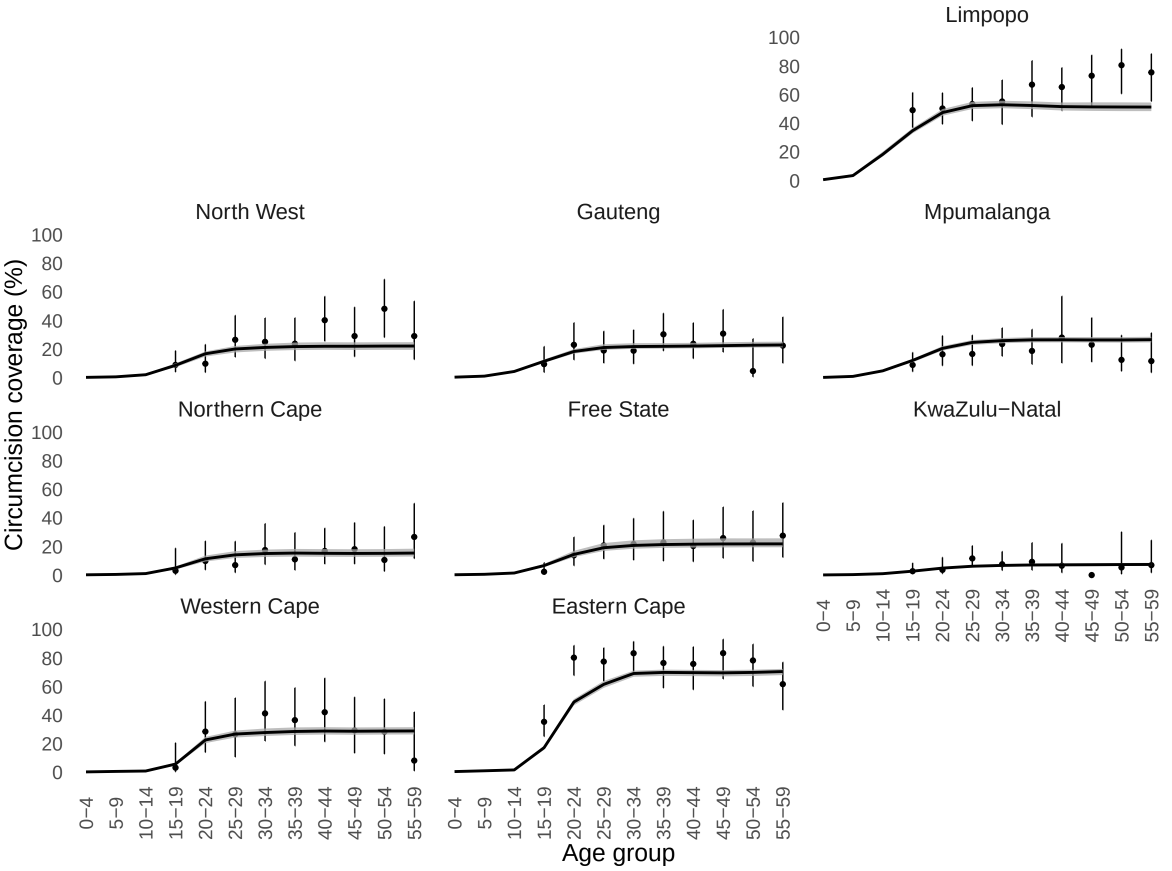

Supplementary Figure 32: Province-level TMIC coverage by 5-year age groups in 2016. Lines denote the estimated mean prevalence, with the shaded regions denoting the 95% CI. Black dots denote the sampled TMIC coverage from the 2016 DHS survey.

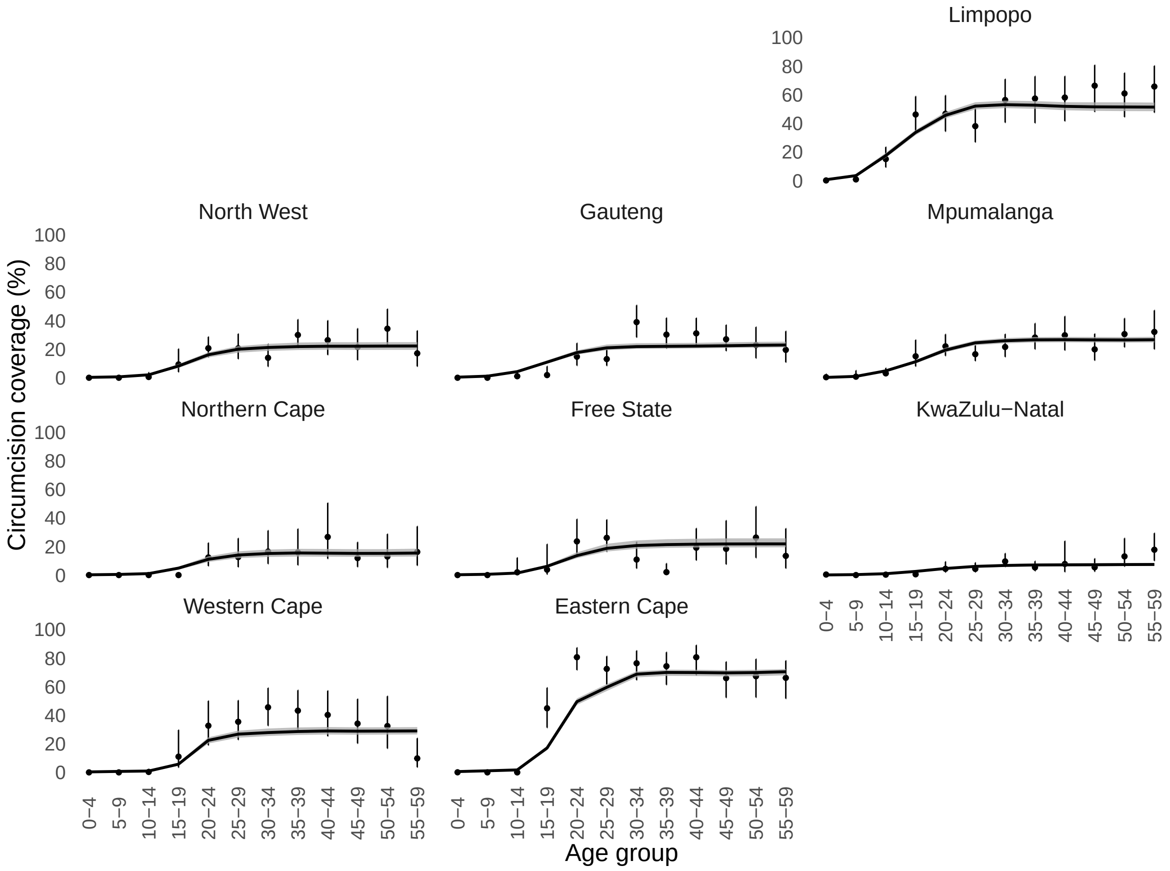

Supplementary Figure 33: Estimated TMIC coverage by 5-year age groups in 2017 at a province level. Lines denote the estimated mean prevalence, with the shaded regions denoting the 95% CI. Black dots denote the sampled TMIC coverage from the 2017 SABSSM survey.

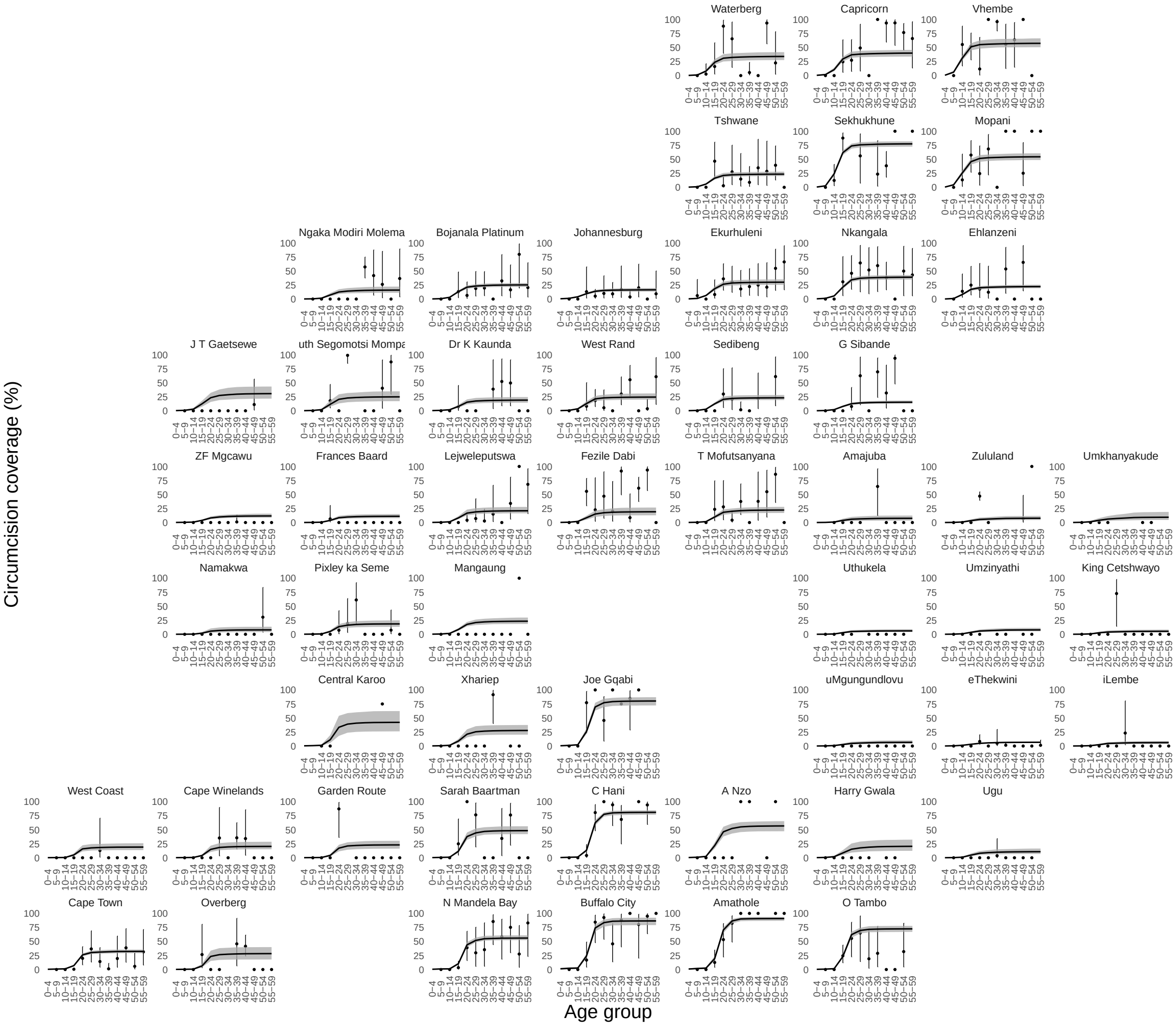

Supplementary Figure 34: District-level TMIC coverage by 5-year age groups in 2002. Lines denote the estimated mean prevalence, with the shaded regions denoting the 95% CI. Black dots denote the sampled TMIC coverage from the 2002 SABSSM survey.

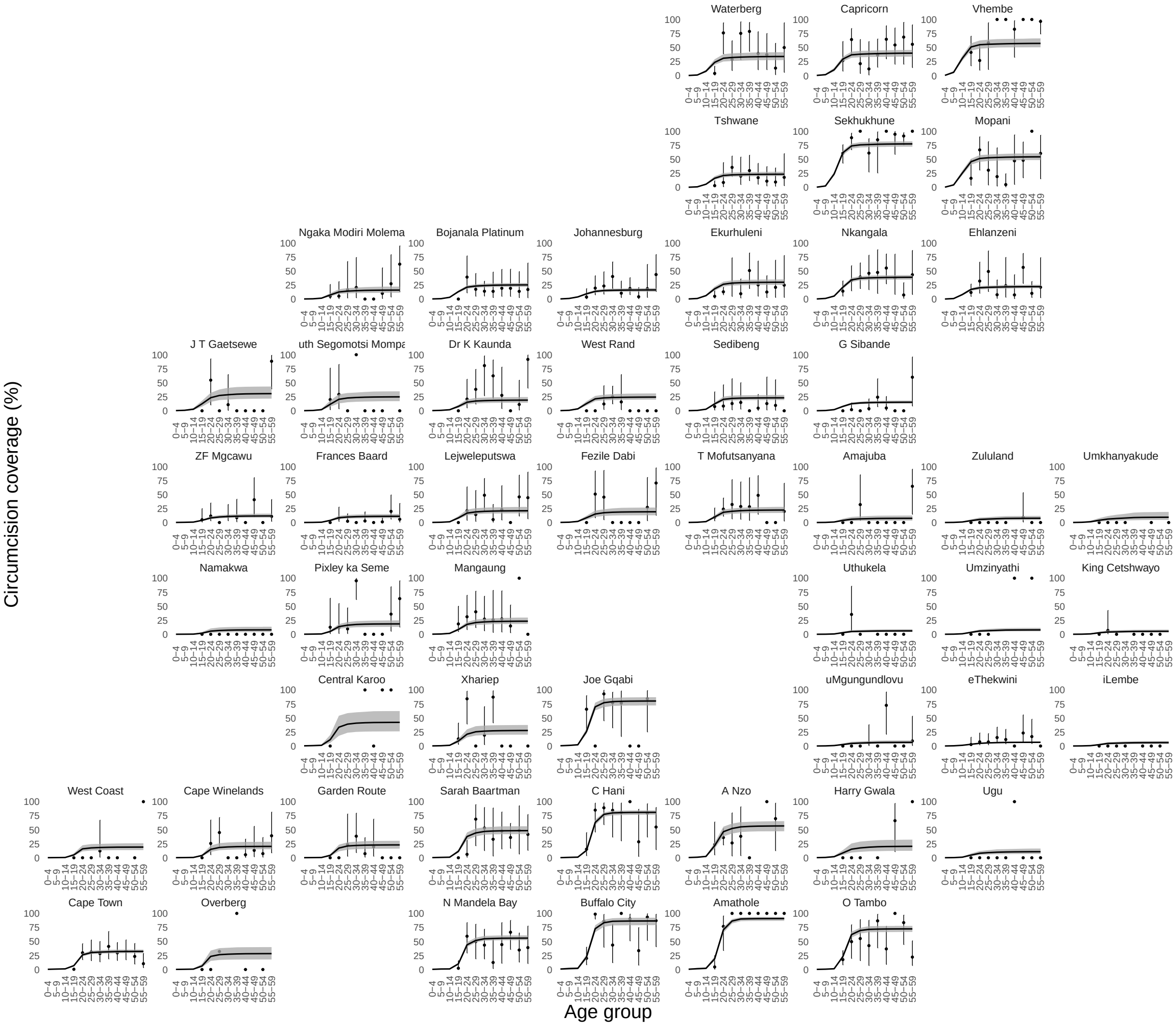

Supplementary Figure 35: District-level TMIC coverage by 5-year age groups in 2008. Lines denote the estimated mean prevalence, with the shaded regions denoting the 95% CI. Black dots denote the sampled TMIC coverage from the 2008 SABSSM survey.

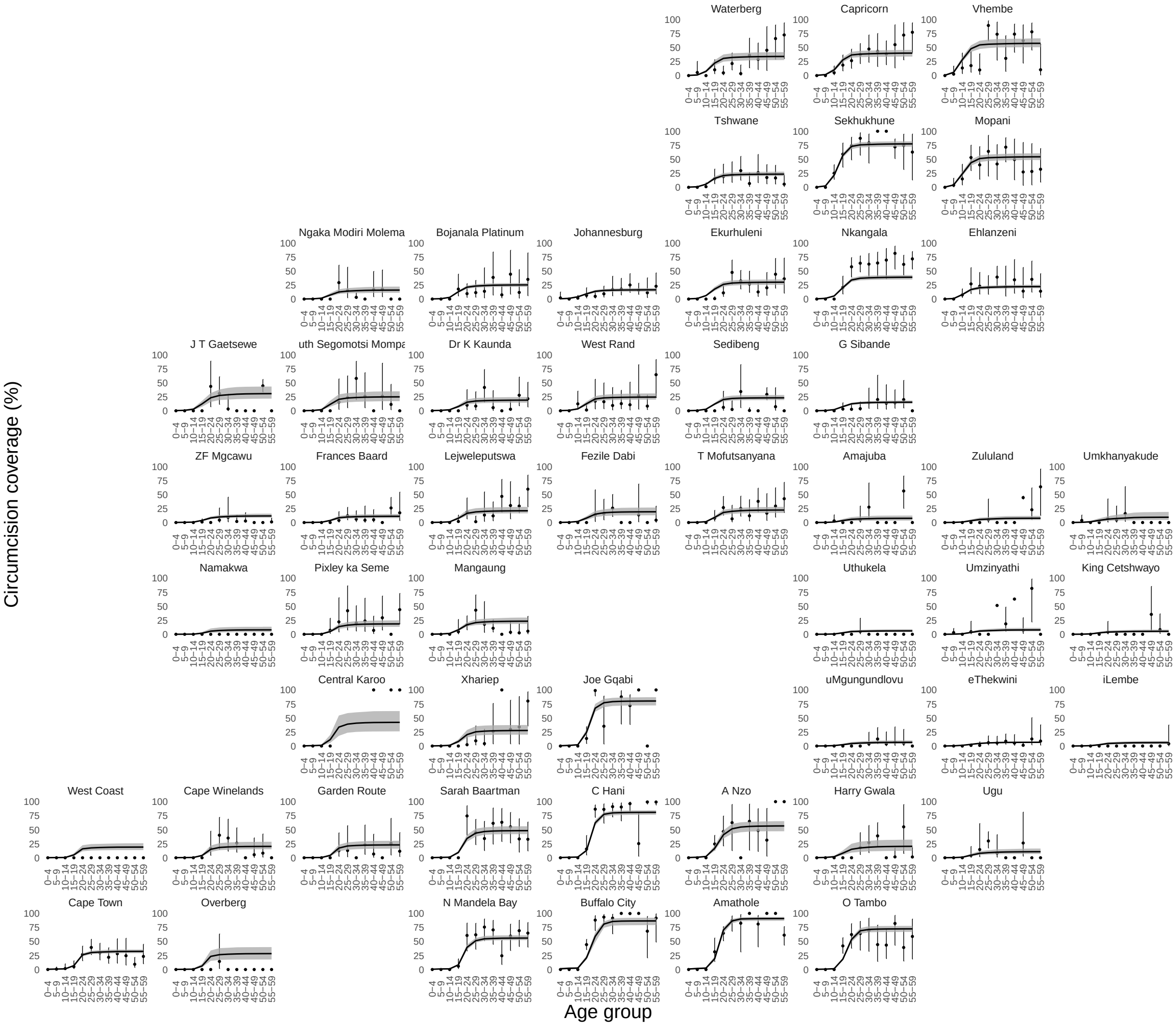

Supplementary Figure 36: District-level TMIC coverage by 5-year age groups in 2012. Lines denote the estimated mean prevalence, with the shaded regions denoting the 95% CI. Black dots denote the sampled TMIC coverage from the 2012 SABSSM survey.

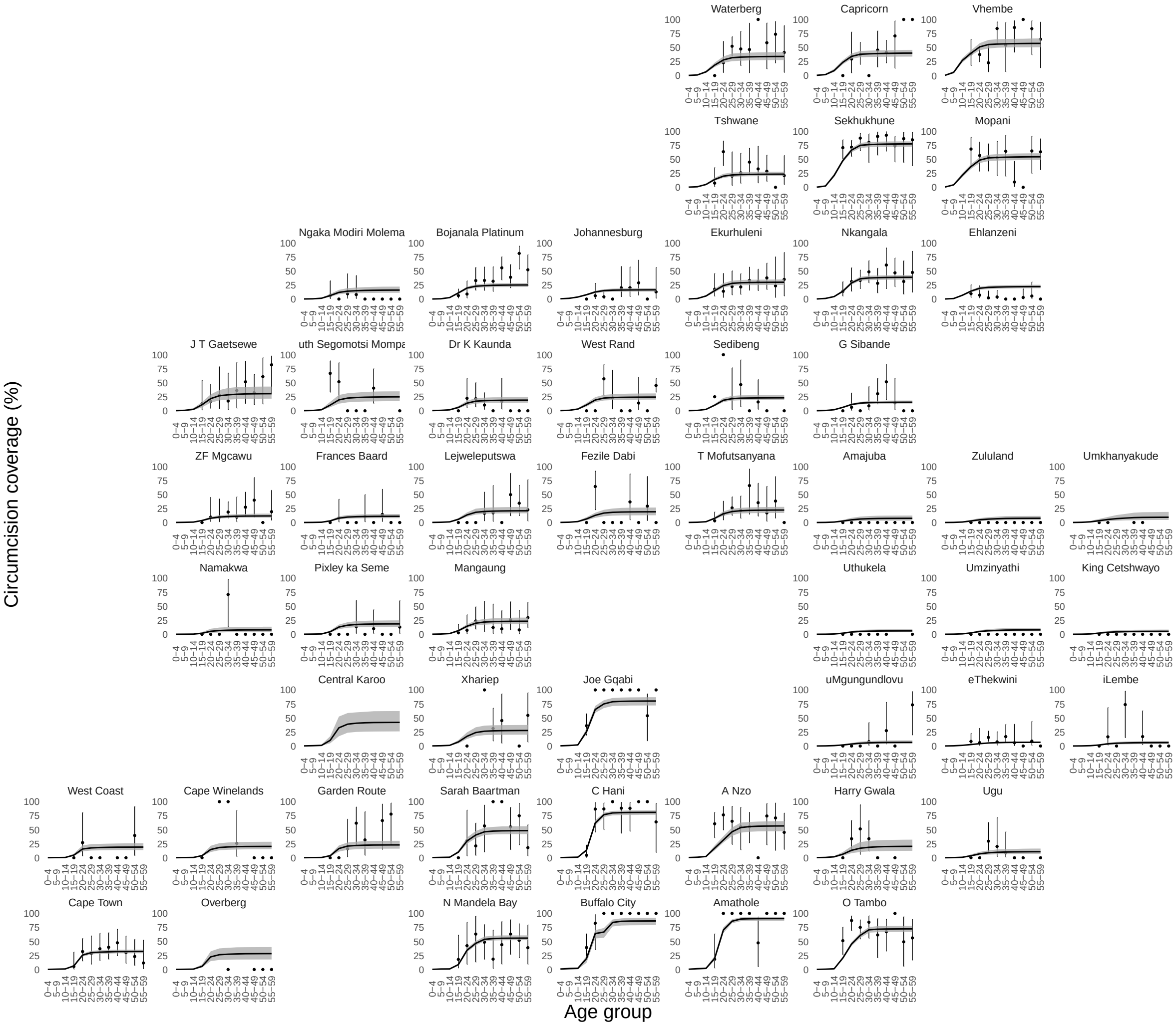

Supplementary Figure 37: Estimated TMIC coverage by 5-year age groups in 2016 at a district level. Lines denote the estimated mean prevalence, with the shaded regions denoting the 95% CI. Black dots denote the sampled TMIC coverage from the 2016 DHS survey.

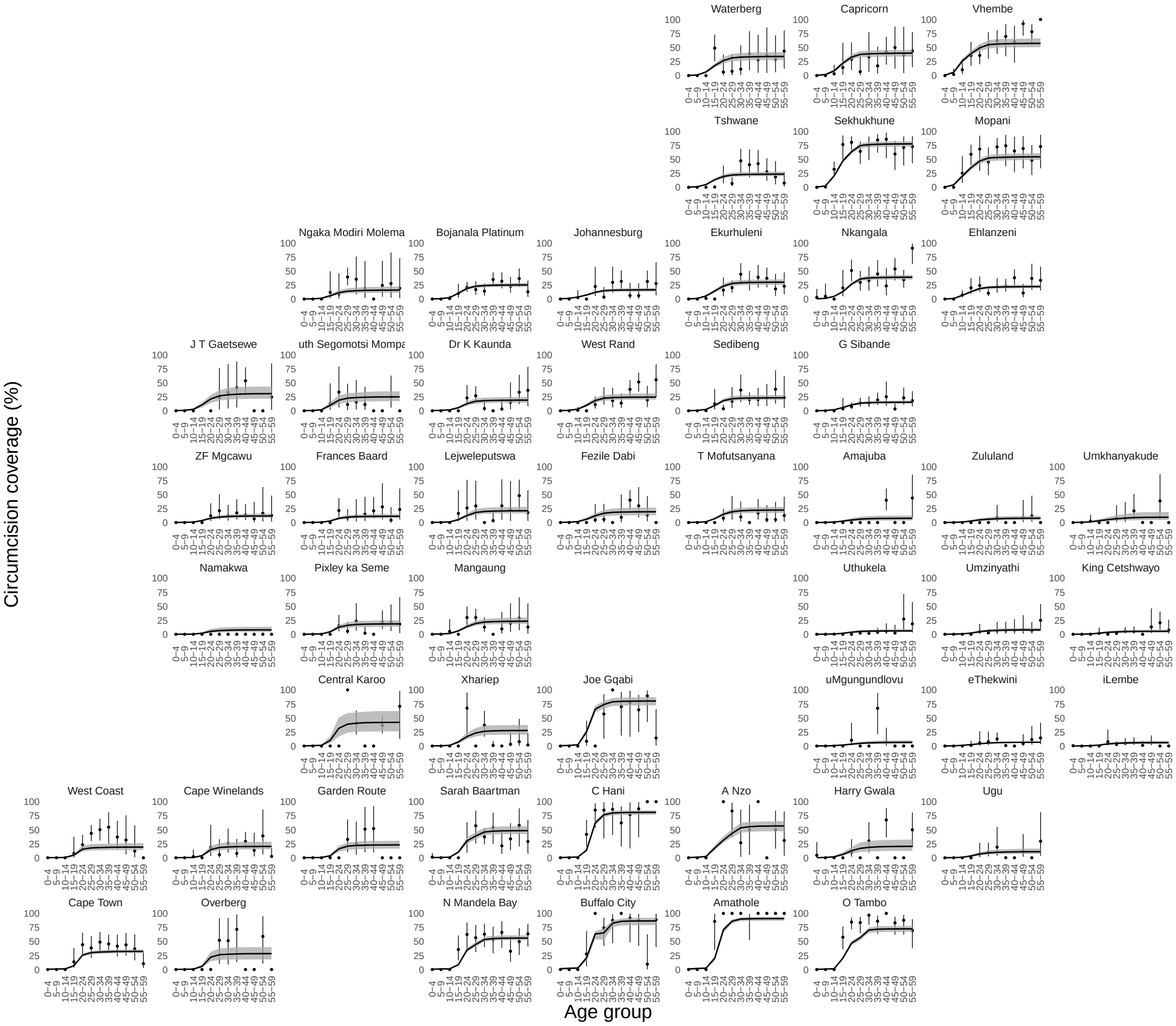

Supplementary Figure 38: District-level TMIC coverage by 5-year age groups in 2017. Lines denote the estimated mean prevalence, with the shaded regions denoting the 95% CI. Black dots denote the sampled TMIC coverage from the 2017 SABSSM survey.

Model fit - Number of VMMCs performed

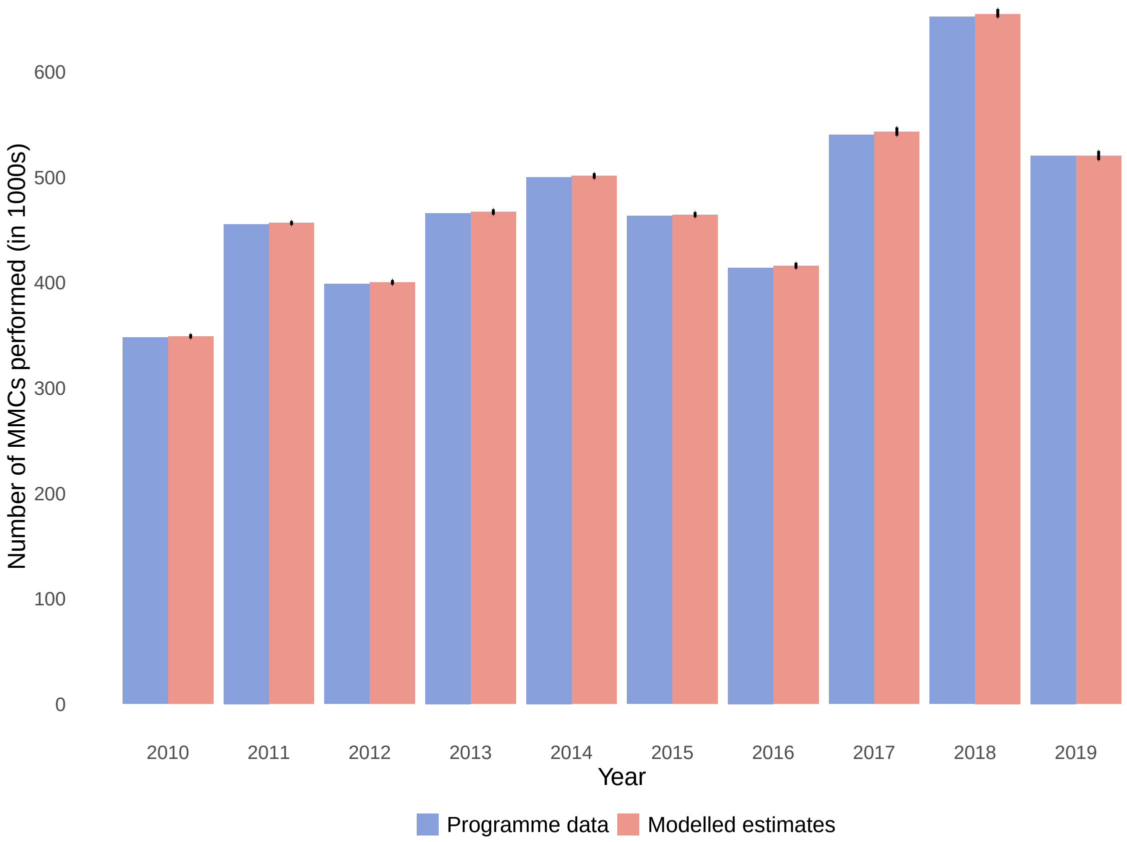

Supplementary Figure 39: Model estimates for number of MMCs conducted annually among men agd 10+ years and the reported number of VMMCs performed nationally between 2010 and 2019.

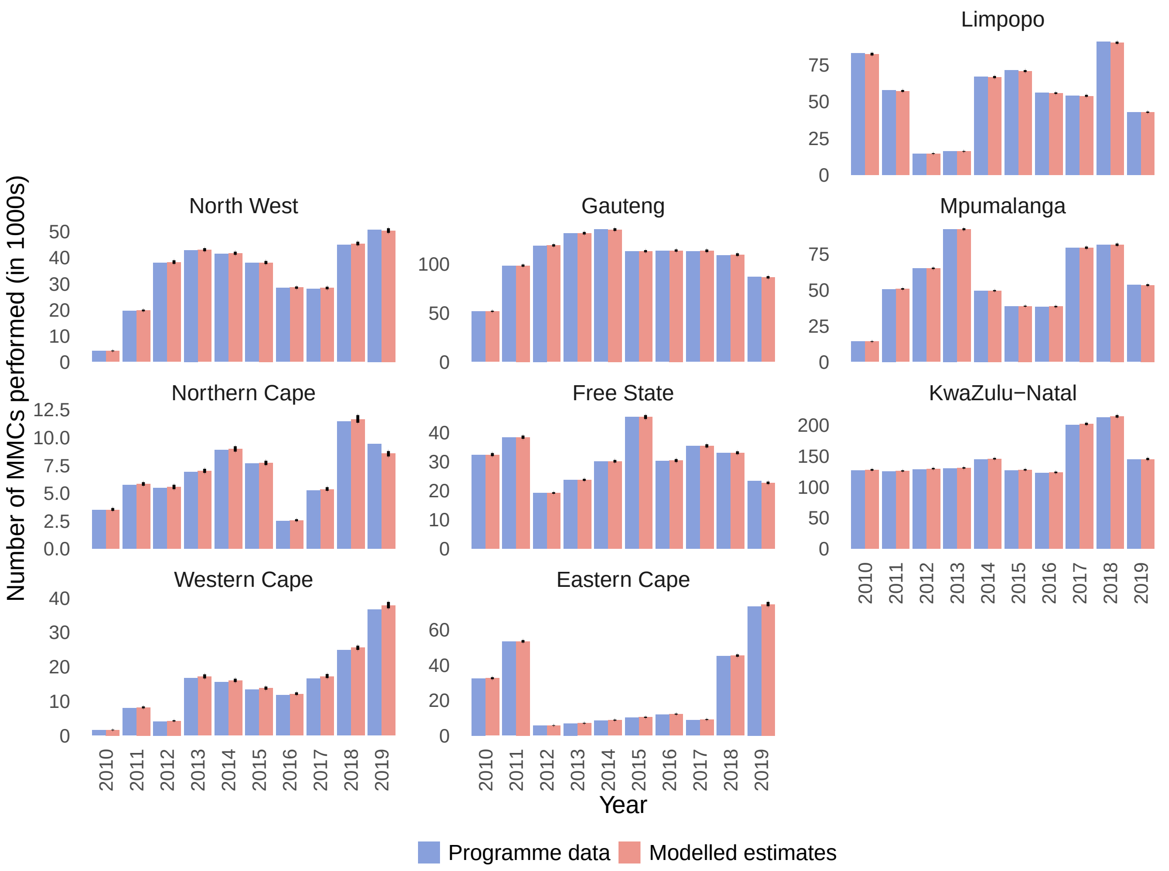

Supplementary Figure 40: Number of MMCs conducted annually among men aged 10+ years and the reported number of VMMCs in each province between 2010 and 2019. Vertical lines denote the 95% CIs.

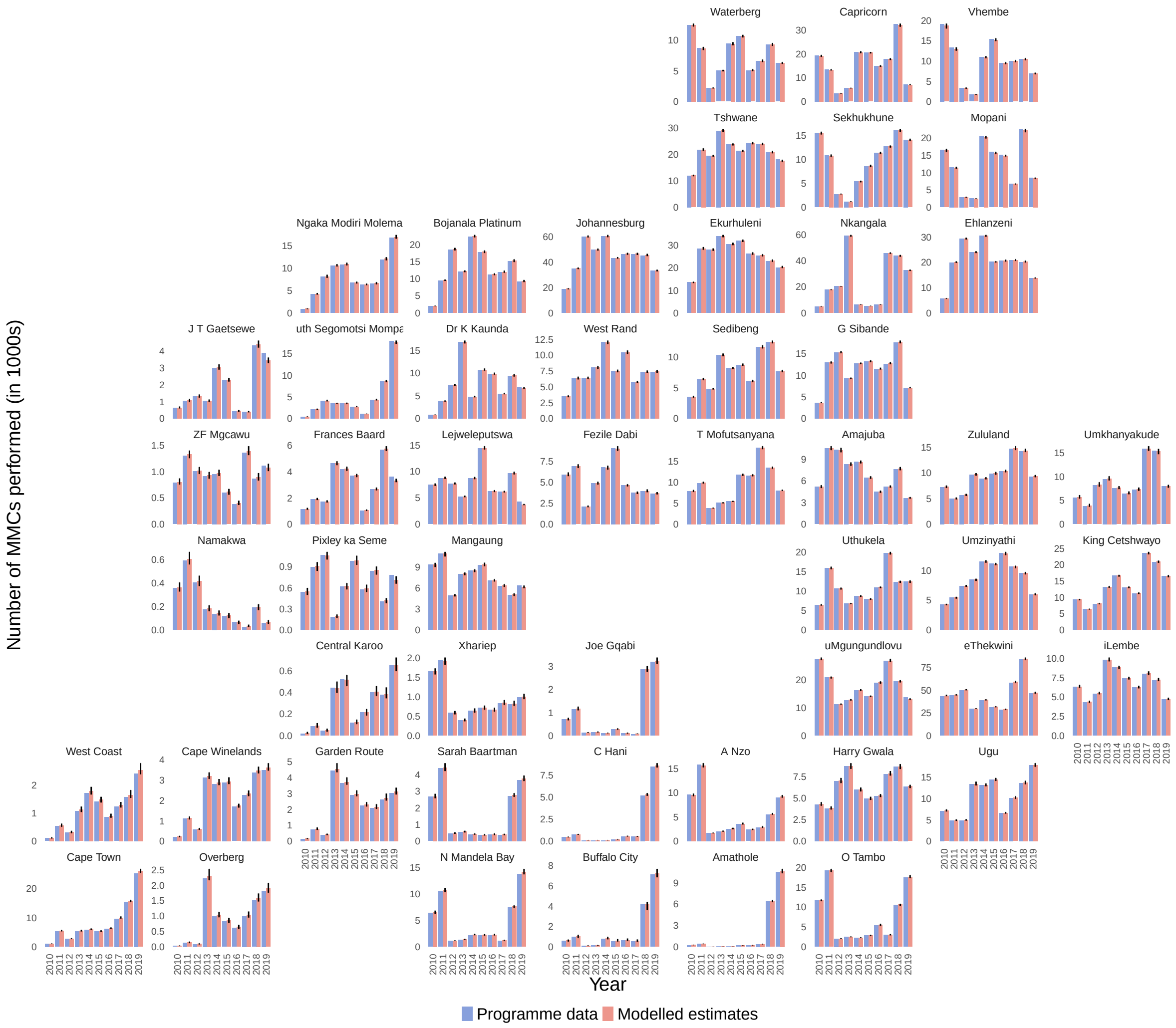

Supplementary Figure 41: Number of MMCs conducted annually among men aged 10+ years and the reported number of VMMCs performed in each district between 2010 and 2019. Vertical lines denote the 95% CIs.

Posterior predictive checks

	Full model						Survey only model					
	**CRPS**	**MAE**	**RMSE**	**50% CI**	**80% CI**	**95% CI**	**CRPS**	**MAE**	**RMSE**	**50% CI**	**80% CI**	**95% CI**
**Overall**	275.00	0.20	0.26	59.48	82.71	94.73	270.80	0.20	0.26	61.02	84.39	95.56
**Survey**												
2002	61.37	0.22	0.32	74.13	91.08	97.90	62.46	0.23	0.32	74.83	91.26	97.55
2008	53.91	0.24	0.32	58.68	86.99	97.49	54.06	0.25	0.32	62.33	87.44	97.26
2012	54.45	0.17	0.21	54.85	77.67	92.88	51.90	0.16	0.21	55.83	81.55	94.34
2016	52.10	0.24	0.30	60.84	82.98	94.64	51.71	0.23	0.30	61.54	83.92	94.41
2017	53.17	0.16	0.20	50.24	76.81	91.79	50.67	0.16	0.19	52.17	79.07	94.52
**Age group**												
0-4	2.50	0.03	0.07	78.06	89.68	98.71	2.49	0.03	0.07	77.42	89.68	98.71
5-9	5.08	0.06	0.09	66.23	83.77	93.51	5.09	0.06	0.09	66.88	83.12	94.16
10-14	10.38	0.12	0.16	52.56	69.87	87.18	8.89	0.11	0.15	53.21	79.49	92.31
15-19	24.18	0.18	0.23	50.98	72.94	90.98	22.37	0.17	0.23	54.90	77.25	92.55
20-24	26.31	0.20	0.25	49.80	79.05	93.28	25.02	0.20	0.25	52.17	83.40	96.84
25-29	24.68	0.21	0.27	61.45	83.53	93.98	24.75	0.21	0.27	61.45	85.54	95.58
30-34	28.00	0.23	0.29	57.85	85.54	96.28	28.01	0.23	0.29	58.68	86.78	95.87
35-39	30.21	0.24	0.31	57.66	84.68	96.37	30.14	0.24	0.31	60.89	85.08	95.56
40-44	30.04	0.24	0.32	59.18	82.86	95.51	29.86	0.24	0.32	63.67	82.45	96.33
45-49	31.45	0.25	0.33	65.06	86.75	96.39	31.66	0.25	0.33	65.86	87.15	95.98
50-54	31.73	0.26	0.34	60.17	84.65	96.27	31.77	0.26	0.34	60.58	84.23	95.02
55-59	30.44	0.26	0.35	62.34	88.31	96.97	30.74	0.27	0.35	62.34	88.74	97.40
15-49	15.73	0.11	0.13	43.24	64.86	83.40	15.01	0.11	0.13	45.17	71.81	87.64

Supplementary Table 4: Results of the posterior predictive checking for total male circumcision (MC) coverage using the full model (including both survey and VMMC programme data) and the model with only household survey data and shown by survey year, age group and overall. For all strata, the continuous ranked probability scores (CRPS), mean absolute error (MAE), root mean square error (RMSE), and the proportion of empirical observations that fell within the 50%, 80%, and 95% quantiles are shown.

	Full model						Survey only model					
	**CRPS**	**MAE**	**RMSE**	**50% CI**	**80% CI**	**95% CI**	**CRPS**	**MAE**	**RMSE**	**50% CI**	**80% CI**	**95% CI**
**Overall**	204.92	0.16	0.22	65.38	85.29	95.03	197.49	0.15	0.22	66.77	87.42	96.45
**Survey**												
2002	35.11	0.15	0.25	78.67	93.88	98.60	35.51	0.15	0.25	79.37	93.53	98.95
2008	36.41	0.17	0.25	71.00	90.64	97.95	36.97	0.18	0.26	69.86	90.41	97.49
2012	43.03	0.14	0.18	59.06	81.23	93.37	39.45	0.13	0.17	62.14	85.44	96.44
2016	42.72	0.20	0.27	66.67	88.34	95.57	41.88	0.20	0.26	67.13	88.58	96.04
2017	47.65	0.14	0.18	54.59	75.52	90.98	43.68	0.14	0.17	57.33	80.84	93.72
**Age group**												
0-4	2.36	0.03	0.06	78.06	87.74	98.06	2.35	0.03	0.06	77.42	88.39	98.06
5-9	5.07	0.06	0.08	64.94	83.12	92.86	5.08	0.06	0.08	64.94	83.12	92.86
10-14	9.62	0.11	0.15	50.64	73.72	85.90	7.84	0.10	0.13	55.13	80.13	96.15
15-19	21.10	0.15	0.20	53.33	75.69	88.63	18.71	0.15	0.20	54.90	80.39	94.51
20-24	20.67	0.16	0.21	57.71	77.87	93.28	18.63	0.15	0.21	59.29	86.17	96.44
25-29	18.16	0.16	0.22	64.26	85.94	95.58	17.01	0.15	0.22	71.49	88.76	96.39
30-34	19.13	0.17	0.24	65.29	89.67	97.52	19.08	0.17	0.23	69.01	90.91	97.93
35-39	22.92	0.19	0.26	68.15	89.92	96.37	22.47	0.18	0.26	70.16	89.52	96.77
40-44	22.95	0.19	0.27	64.90	88.16	97.55	22.81	0.19	0.26	66.94	88.98	96.73
45-49	21.81	0.19	0.27	72.29	88.35	96.79	21.72	0.18	0.27	69.88	89.16	96.39
50-54	21.32	0.19	0.27	70.95	87.97	98.34	21.66	0.19	0.27	70.12	89.21	97.93
55-59	19.83	0.19	0.28	74.46	92.21	96.97	20.14	0.19	0.28	71.86	90.91	96.54
15-49	12.99	0.09	0.11	42.47	66.80	81.08	11.58	0.09	0.11	45.95	71.43	89.19

Supplementary Table 5: Results of the posterior predictive checking for coverage in medical male circumcision for non-traditional reasons (MMC-nT) using the full model (including both survey and VMMC programme data) and the model with only household survey data and shown by survey year, age group and overall. For all strata, the continuous ranked probability scores (CRPS), mean absolute error (MAE), root mean square error (RMSE), and the proportion of empirical observations that fell within the 50%, 80%, and 95% quantiles are shown.

	Full model						Survey only model					
	**CRPS**	**MAE**	**RMSE**	**50% CI**	**80% CI**	**95% CI**	**CRPS**	**MAE**	**RMSE**	**50% CI**	**80% CI**	**95% CI**
**Overall**	210.74	0.15	0.21	68.60	86.63	95.56	207.87	0.15	0.21	68.56	86.63	95.97
**Survey**												
2002	46.21	0.17	0.25	80.59	93.53	98.95	46.24	0.17	0.25	80.77	93.18	98.43
2008	46.12	0.20	0.28	64.84	87.67	96.58	46.41	0.20	0.28	63.70	87.44	97.03
2012	39.97	0.12	0.16	64.56	85.44	94.66	38.95	0.12	0.16	65.53	84.47	95.47
2016	39.24	0.18	0.24	69.93	85.55	94.64	38.33	0.18	0.24	69.70	86.48	94.87
2017	39.20	0.12	0.15	63.29	81.48	93.24	37.94	0.12	0.15	62.96	82.29	94.20
**Age group**												
00-04	0.22	0.00	0.02	95.48	96.13	99.35	0.21	0.00	0.02	95.48	96.13	99.35
05-09	0.41	0.01	0.03	96.10	96.75	99.35	0.42	0.01	0.03	94.16	96.75	100.00
10-14	1.63	0.03	0.05	82.69	94.23	98.72	1.69	0.03	0.05	83.33	91.03	98.72
15-19	12.51	0.10	0.14	67.45	85.88	92.16	12.07	0.10	0.14	68.24	86.27	93.73
20-24	23.59	0.17	0.21	53.36	79.84	90.91	22.23	0.16	0.21	54.55	80.24	92.89
25-29	20.38	0.17	0.23	65.86	85.54	94.78	19.82	0.17	0.22	64.66	86.35	94.78
30-34	23.18	0.19	0.25	64.05	85.12	94.63	23.42	0.19	0.25	63.64	86.36	95.04
35-39	24.04	0.19	0.26	65.73	86.69	96.37	23.90	0.19	0.26	66.94	86.29	96.37
40-44	25.72	0.20	0.27	60.82	80.41	95.92	25.48	0.20	0.27	62.45	82.45	95.10
45-49	24.15	0.20	0.28	68.27	88.35	96.39	23.98	0.20	0.28	69.48	87.15	97.19
50-54	28.84	0.22	0.30	61.83	85.89	97.10	28.71	0.22	0.30	58.92	85.06	96.68
55-59	26.08	0.22	0.30	67.10	84.85	95.24	25.95	0.22	0.30	65.80	84.42	95.67
15-49	12.49	0.09	0.11	47.49	72.20	89.19	11.82	0.09	0.10	47.88	73.36	89.19

Supplementary Table 6: Results of the posterior predictive checking for coverage in male circumcision conducted in male initiation ceremonies (TMIC) using the full model (including both survey and VMMC programme data) and the model with only household survey data and shown by survey year, age group and overall. For all strata, the continuous ranked probability scores (CRPS), mean absolute error (MAE), root mean square error (RMSE), and the proportion of empirical observations that fell within the 50%, 80%, and 95% quantiles are shown.

Supplementary References

1. Putter, H., Fiocco, M. & Geskus, R. B. Tutorial in biostatistics: Competing risks and multi-state models. *Statistics in Medicine* **26,** 2389–2430 (2007).
2. Besag, J. & Kooperberg, C. On conditional and intrinsic autoregressions. *Biometrika* **82,** 733–746 (1995).
3. Rue, H. & Held, L. *Gaussian Markov random fields: theory and applications* (CRC press, 2005).
4. Knorr-Held, L. Bayesian modelling of inseparable space-time variation in disease risk. *Statistics in medicine* **19,** 2555–2567 (2000).
5. R Core Team. R: A Language and Environment for Statistical Computing. *R Foundation for Statistical Computing, Vienna, Austria.* https://www.R-project.org/ (2021).
6. Kristensen, K., Nielsen, A., Berg, C. W., Skaug, H. & Bell, B. M. TMB: Automatic differentiation and Laplace approximation. *Journal of Statistical Software* **70,** 1–21 (2016).
7. Byrd, R. H., Lu, P., Nocedal, J. & Zhu, C. A limited memory algorithm for bound constrained optimization. *SIAM Journal on scientific computing* **16,** 1190–1208 (1995).
8. Eaton, J. W. *et al.* Naomi: A New Modelling Tool for Estimating HIV Epidemic Indicators at the District Level in Sub-Saharan Africa. *Under review* (2021).
