# Supplementary material for: Substantial but spatially heterogeneous progress in male circumcision for HIV prevention in South Africa
Source: Commun Med (Lond). 2024 Jan 3;4:1. doi: 10.1038/s43856-023-00405-7 (PMC10764768; doi:10.1038/s43856-023-00405-7)
Supplement: Supplementary file 12 — Reporting Summary [file 43856_2023_405_MOESM12_ESM.pdf]

nature portfolio

Corresponding author(s): Dr Matthew L. Thomas

Last updated by author(s): Sep 25, 2023

Reporting Summary

Nature Portfolio wishes to improve the reproducibility of the work that we publish. This form provides structure for consistency and transparency in reporting. For further information on Nature Portfolio policies, see our Editorial Policies and the Editorial Policy Checklist.

Statistics

For all statistical analyses, confirm that the following items are present in the figure legend, table legend, main text, or Methods section.

n/a	Confirmed	
☒	☐	The exact sample size (*n*) for each experimental group/condition, given as a discrete number and unit of measurement
☒	☐	A statement on whether measurements were taken from distinct samples or whether the same sample was measured repeatedly
☒	☐	The statistical test(s) used AND whether they are one- or two-sided<br>*Only common tests should be described solely by name; describe more complex techniques in the Methods section.*
☒	☐	A description of all covariates tested
☒	☐	A description of any assumptions or corrections, such as tests of normality and adjustment for multiple comparisons
☐	☒	A full description of the statistical parameters including central tendency (e.g. means) or other basic estimates (e.g. regression coefficient) AND variation (e.g. standard deviation) or associated estimates of uncertainty (e.g. confidence intervals)
☒	☐	For null hypothesis testing, the test statistic (e.g. *F*, *t*, *r*) with confidence intervals, effect sizes, degrees of freedom and *P* value noted<br>*Give P values as exact values whenever suitable.*
☐	☒	For Bayesian analysis, information on the choice of priors and Markov chain Monte Carlo settings
☒	☐	For hierarchical and complex designs, identification of the appropriate level for tests and full reporting of outcomes
☒	☐	Estimates of effect sizes (e.g. Cohen's *d*, Pearson's *r*), indicating how they were calculated

Our web collection on statistics for biologists contains articles on many of the points above.

Software and code

Policy information about availability of computer code

Data collection	The software R was used to perform data analysis, implement the models and produce results presented in this manuscript. The code is available from https://github.com/mrc-ide/zaf-circumcision-rates. An R package implementing the model is available from https://github.com/mrc-ide/threemc.
Data analysis	The software R was used to perform data analysis, implement the models and produce results presented in this manuscript. The code is available from https://github.com/mrc-ide/zaf-circumcision-rates. An R package implementing the model is available from https://github.com/mrc-ide/threemc.

For manuscripts utilizing custom algorithms or software that are central to the research but not yet described in published literature, software must be made available to editors and reviewers. We strongly encourage code deposition in a community repository (e.g. GitHub). See the Nature Portfolio guidelines for submitting code & software for further information.

Data

Policy information about availability of data

All manuscripts must include a data availability statement. This statement should provide the following information, where applicable:

- Accession codes, unique identifiers, or web links for publicly available datasets
- A description of any restrictions on data availability
- For clinical datasets or third party data, please ensure that the statement adheres to our policy

Household survey data are available by request to the South Africa Human Science Research Council (HSRC; http://datacuration.hsrc.ac.za/content/view/access-to-data) and The Demographic and Health Survey (DHS) Program (https://dhsprogram.com/methodology/survey/survey-display-390.cfm). Information on the number of VMMCs performed were obtained from DMPPT2 and South Africa National Department of Health (NDoH) DHIS are by data request to the South Africa NDoH. Data on VMMCs conducted from PEPFAR MER are available from https://data.pepfar.gov/datasets#PDD. Estimates of population were obtained from Thembisa and Statistics South Africa, which are available from https://thembisa.org/downloads and http://www.statssa.gov.za/?page_id=1854&PPN=P0302&SCH=72634, respectively. Estimates of the proportions of men by primary language group in South Africa were obtained from the South Africa 2011 census, which is available from https://www.datafirst.uct.ac.za/dataportal/index.php/catalog/485.

Human research participants

Policy information about studies involving human research participants and Sex and Gender in Research.

Reporting on sex and gender	This study reports on male circumcision coverage by district, age, and type of circumcision. Therefore study results apply to only male sex.
Population characteristics	Study participants are male survey respondents of all ages from nationally-representative household surveys in South Africa.
Recruitment	Study participants were recruited through household visits involving household enumeration followed by invitation to participate in the household surveys.
Ethics oversight	HSRC's Research Ethics Committee; CDC IRB; SA MRC Research Ethics Committee; ICF Macro IRB

Note that full information on the approval of the study protocol must also be provided in the manuscript.

Field-specific reporting

Please select the one below that is the best fit for your research. If you are not sure, read the appropriate sections before making your selection.

☐ Life sciences ☒ Behavioural & social sciences ☐ Ecological, evolutionary & environmental sciences

For a reference copy of the document with all sections, see nature.com/documents/nr-reporting-summary-flat.pdf

Behavioural & social sciences study design

All studies must disclose on these points even when the disclosure is negative.

Study description	This study involv
Research sample	This study involves secondary data about (1) male respondents to five nationally representative household surveys conducted in South Africa between 2002 and 2017, and (2) national HIV programme data about the number of voluntary medical male circumcisions conducted for HIV prevention from 2008 to 2019.
Sampling strategy	Nationally representative household surveys were sampled via a two-stage stratified sampling strategy. Samples were stratified according to province and residence type (urban / rural; or urban / traditional / farms).
Data collection	Data collection procedures varied across household surveys between pen and paper and tablet-based data collection. Where possible, no other individuals were present other than the respondent and the interviewer to ensure confidentiality of responses. The survey did not involve any experimental condition.
Timing	Household survey data used in this study were collected in 2002, 2008, 2012, 2016, and 2017.
Data exclusions	Female survey participants were excluded because the study pertained to male circumcision. This was predetermined.
Non-participation	Details on non-participation in each survey are available from primary survey reports.
Randomization	This study did not involve any experimental groups.

Reporting for specific materials, systems and methods

We require information from authors about some types of materials, experimental systems and methods used in many studies. Here, indicate whether each material, system or method listed is relevant to your study. If you are not sure if a list item applies to your research, read the appropriate section before selecting a response.

Materials & experimental systems

n/a	Involved in the study
☒	☐ Antibodies
☒	☐ Eukaryotic cell lines
☒	☐ Palaeontology and archaeology
☒	☐ Animals and other organisms
☒	☐ Clinical data
☒	☐ Dual use research of concern

Methods

n/a	Involved in the study
☒	☐ ChIP-seq
☒	☐ Flow cytometry
☒	☐ MRI-based neuroimaging
